# Distribution and Phylogeny of Microsymbionts Associated with Cowpea (Vigna unguiculata) Nodulation in Three Agroecological Regions of Mozambique

**DOI:** 10.1128/AEM.01712-17

**Published:** 2018-01-02

**Authors:** Ifeoma N. Chidebe, Sanjay K. Jaiswal, Felix D. Dakora

**Affiliations:** a Department of Crop Sciences, Tshwane University of Technology, Pretoria, South Africa; b Department of Chemistry, Tshwane University of Technology, Arcadia Campus, Pretoria, South Africa; Goethe University Frankfurt am Main

**Keywords:** BOX-PCR, ITS, phylogeny, nodulation, horizontal gene transfer, metagenome, Bradyrhizobium, Rhizobium, agroecology

## Abstract

Cowpea derives most of its N nutrition from biological nitrogen fixation (BNF) via symbiotic bacteroids in root nodules. In Sub-Saharan Africa, the diversity and biogeographic distribution of bacterial microsymbionts nodulating cowpea and other indigenous legumes are not well understood, though needed for increased legume production. The aim of this study was to describe the distribution and phylogenies of rhizobia at different agroecological regions of Mozambique using PCR of the BOX element (BOX-PCR), restriction fragment length polymorphism of the internal transcribed spacer (ITS-RFLP), and sequence analysis of ribosomal, symbiotic, and housekeeping genes. A total of 122 microsymbionts isolated from two cowpea varieties (IT-1263 and IT-18) grouped into 17 clades within the BOX-PCR dendrogram. The PCR-ITS analysis yielded 17 ITS types for the bacterial isolates, while ITS-RFLP analysis placed all test isolates in six distinct clusters (I to VI). BLAST_n_ sequence analysis of 16S rRNA and four housekeeping genes (*glnII*, *gyrB*, *recA*, and *rpoB*) showed their alignment with Rhizobium and Bradyrhizobium species. The results revealed a group of highly diverse and adapted cowpea-nodulating microsymbionts which included Bradyrhizobium pachyrhizi, Bradyrhizobium arachidis, Bradyrhizobium yuanmingense, and a novel Bradyrhizobium sp., as well as Rhizobium tropici, Rhizobium pusense, and Neorhizobium galegae in Mozambican soils. Discordances observed in single-gene phylogenies could be attributed to horizontal gene transfer and/or subsequent recombinations of the genes. Natural deletion of 60 bp of the *gyrB* region was observed in isolate TUTVU7; however, this deletion effect on DNA gyrase function still needs to be confirmed. The inconsistency of *nifH* with core gene phylogenies suggested differences in the evolutionary history of both chromosomal and symbiotic genes.

**IMPORTANCE** A diverse group of both Bradyrhizobium and Rhizobium species responsible for cowpea nodulation in Mozambique was found in this study. Future studies could prove useful in evaluating these bacterial isolates for symbiotic efficiency and strain competitiveness in Mozambican soils.

## INTRODUCTION

For millennia, humans have utilized legumes as a source of food, animal fodder, traditional medicine, shelter, and fuel (1). In Africa's diverse cultures, grain legumes constitute an integral component of cropping systems, especially in the rural communities. In Mozambique, for example, cowpea is the most important food grain legume, grown extensively on smallholder fields and estimated at nearly four million hectares in land area. The high protein content of cowpea grain and leaves (2) can augment the low-protein diets of rural Mozambican households, with potential to eliminate protein calorie malnutrition. Cowpea grain also contains 57% carbohydrates (2), essential amino acids, dietary fiber, and abundant minerals (3) and thus contributes to a balanced diet.

In addition to their nutritional importance, legumes are very highly valued in the agroecosystem as they have the most efficient biological nitrogen fixation (BNF) system known in nature (4). BNF in legumes occurs through a symbiotic partnership between the host plant and soil bacteria collectively known as rhizobia, which provide biologically fixed atmospheric N to the legume in exchange for plant photosynthate. The distinctive feature of this partnership is the presence of specialized symbiotic organs, or nodules, which are developed on the roots and occasionally stems of legumes following a series of molecular exchanges and morphological modifications in the two partners (5).

Legume fixed N can meet more than half of the total N needs of a legume (6). As a result, plant growth and productivity are often less affected by soil N deficiency (7). Through N contribution in cropping systems, the legume/rhizobium symbiosis has the potential to promote and sustain agricultural productivity, particularly in the low-input systems of Sub-Saharan Africa (8).

Although they all are capable of forming root or stem nodules, rhizobia can be phylogenetically and metabolically very diverse (4). Currently, there are about 15 genera of symbiotic N_2_-fixing bacteria belonging to the phylum Proteobacteria in the alpha, beta, and gamma classes. Representative N_2_ fixers are found in the genera Agrobacterium, Allorhizobium, Azorhizobium, Bradyrhizobium, Burkholderia, Cupriavidus, Devosia, Herbaspirrilum, Mesorhizobium, Methylobacterium, Ochrobactrum, Phyllobacterium, Rhizobium, Shinella, and Sinorhizobium (9). To date, more than 113 nodulating species have been identified within these genera. However, knowledge gaps still exist regarding the genetic diversity and biogeographic distribution of the N_2_-fixing microsymbionts of many legumes (10), especially in Sub-Saharan Africa, where the bacterial symbionts have not been studied for many indigenous legumes. This therefore emphasizes the need to assess the rhizobial genetic diversity within the region and determine the taxonomic affiliations of the bacteria nodulating native legumes.

A determination of the taxonomy and phylogeny of rhizobia is key to improving legume productivity via BNF in diverse environments. Not only has this increased our understanding of the diversity and genetic relatedness of rhizobia, but it also has significantly improved rhizobial classification through (i) the redefinition of species lineages (10, 11), (ii) the identification of rhizobia from more genera of alpha- and betaproteobacteria (12–14), and (iii) the discovery of novel species (15). Such studies are important for generating a genetic resource base from which highly adapted and efficient rhizobial strains are selected for inoculant production (15). Therefore, well-tested, quick methods are required for differentiating rhizobial isolates (16). PCR of the BOX element (BOX-PCR) and PCR-restriction fragment length polymorphism (RFLP) analyses have become powerful tools for detecting diversity among rhizobial isolates at the genome level (16, 17).

Sequencing of the 16S rRNA gene, which is functionally conserved and universally distributed, has become one of the most important tools for defining kingdom and genera but not species (18, 19). To overcome this, the 16S rRNA phylogenetic analysis is usually complemented with data from more rapidly evolving loci, such as the protein-coding housekeeping genes. These genes have a much higher level of sequence divergence, especially in relation to the 16S rRNA gene, but are sufficiently well conserved to retain genetic information for characterizing bacteria at the intra- and interspecific levels (20). Previous studies of cowpea rhizobia have reported nodulation by different species in the genera Bradyrhizobium, Rhizobium, Sinorhizobium, Ralstonia, Achromobacter, and Microvirga (7, 15, 21–24). Clearly, this suggests that the phylogeny of rhizobia nodulating cowpea is still unclear and requires further study (22, 25).

Cowpea is cultivated in all the 10 agroecological zones (AEZs) of Mozambique and contributes significantly to protein calorie requirements in rural households. However, to date, little information exists on the diversity and phylogeny of cowpea-nodulating bacteria in many African countries, including Mozambique. The aim of this study was to determine the diversity and phylogenetic relationships of cowpea rhizobia isolated from experimental sites located in three different agroecological regions of Mozambique using BOX-PCR and RFLP of the internal transcribed spacer (ITS-RFLP) analyses and sequence analysis of 16S rRNA, *nifH*, and housekeeping genes (*recA*, *glnII*, *gyrB*, and *recA*).

## RESULTS

### Bacterial isolation from root nodules.

A total of 122 bacterial isolates were obtained from root nodules of both cowpea varieties (IT-1263 and IT-18) planted at Muriaze, Ruace, and Sussundenga in Mozambique. Of the 122 isolates, 39 originated from Muriaze, 42 from Ruace and 41 from Sussundenga.

### PCR amplification of the repetitive (rep, i.e., BOX) genomic region.

**FIG 1:**
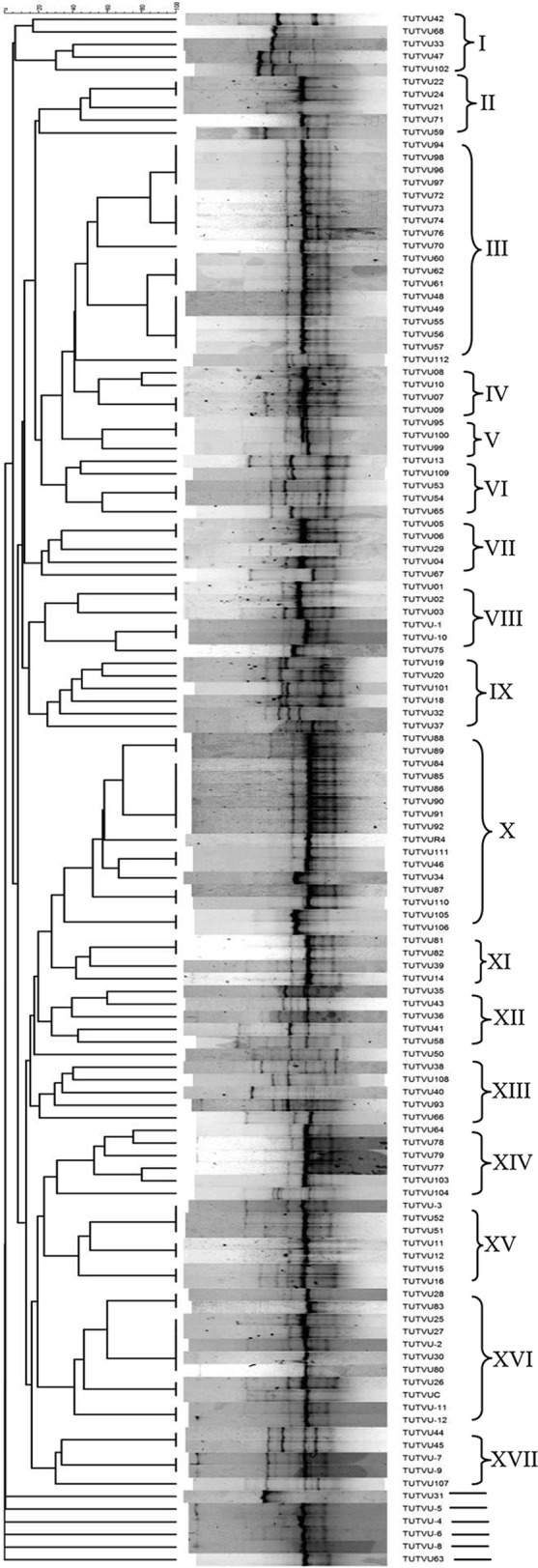
Dendrogram based on BOX-PCR fingerprints of cowpea nodule isolates.

PCR amplification of the BOX region of the genomic DNA from each isolate resulted in distinctive banding patterns, ranging from one to six fragments per DNA profile. Most of the bands were very clear; however, a few faint bands were also observed. The cluster analysis (unweighted pair group method using average linkages [UPGMA] algorithm and Jaccard's similarity coefficient) differentiated the bacterial isolates into 17 clades at 50% Jaccard's similarity coefficient (Fig. 1). The number of isolates in each clade ranged from 2 to 17, with six isolates (TUTVU31, TUTVU-5, TUTVU-4, TUTVU-6, TUTVU-8, and TUTVU63) showing high genetic distinctiveness from the others at 0% Jaccard's similarity coefficient. Conversely, there was 100% similarity coefficient for 68 bacterial isolates (55%), and these were distributed within 9 of the 17 clades. Interestingly, among these, only isolate TUTVU26 showed 100% Jaccard's similarity coefficient with the commercial inoculant BIOFIX (TUTVC) (Fig. 1). There was no clear distinction based on isolate site of origin, as the rhizobial isolates from the three study sites were distributed across all the clades, except for clade XII, which consisted of bacterial isolates exclusively from the Ruace site (Table 1; Fig. 1). Isolates from Muriaze were distributed across 11 different clades, while those from Ruace and Sussundenga were found in 12 and 13 different clades, respectively.

**TABLE 1:** Origin, BOX-PCR, ITS (16S-23S rRNA) characterization, nodulation status and colony morphology of symbiotic bacteria isolated from the root nodules of cowpea from Mozambique^*a*^

Experimental site	Isolate	Cowpea variety	BOX-PCR cluster	ITS band size (bp)	ITS type	ITS-RFLP type (HaeII, HindIII, HinfI)	Colony morphology			Nodulation assay
							Days to emergence	Color	Size (mm)	
Muriaze	TUTVU1	IT-18	VIII	1,000	IX	AA-	5–7	Milky	1.2	+
	TUTVU2	IT-18	VIII	1,107	X	BBA	5–7	White	0.9	+
	TUTVU3	IT-1263	VIII	1,000	IX	CCB	5–7	Milky	1.0	+
	TUTVU4	IT-18	VII	500	II	DDC	5–7	Milky	1.0	+
	TUTVU5	IT-18	VII	1,000	IX	EED	5–7	Milky	1.0	+
	TUTVU6	IT-1263	VII	1,000	IX	EED	5–7	Milky	1.1	+
	TUTVU7	IT-18	IV	1,000	IX	FCD	5–7	Milky	1.0	+
	TUTVU8	IT-18	IV	500	II	DDE	7–10	White	1.0	+
	TUTVU9	IT-1263	V	1,000	IX	AAD	5–7	White	0.8	+
	TUTVU10	IT-1263	IV	1,000	IX	CCD	5–7	Milky	0.8	+
	TUTVU11	IT-18	XV	1,000	IX	CCD	5–7	Translucent	0.9	+
	TUTVU12	IT-1263	XV	1,000	IX	AAD	5–7	Translucent	0.9	+
	TUTVU13	IT-1263	VI	1,000	IX	AAD	5–7	White	0.9	+
	TUTVU14	IT-18	XI	1,000	IX	CCD	7–10	Milky	0.7	+
	TUTVU15	IT-18	XV	1,000	IX	GCD	7–10	Milky	0.8	+
	TUTVU16	IT-1263	XV	1,000	IX	GCD	5–7	Milky	0.8	+
	TUTVU17	IT-1263	X	1,000, 500	XVI	HED	5–7	Milky	0.6	+
	TUTVU18	IT-18	IX	1,000	IX	EAD	7–10	Milky	0.7	+
	TUTVU19	IT-1263	IX	1,000	IX	EAD	5–7	Milky	1.2	+
	TUTVU20	IT-1263	IX	1,000	IX	EAD	5–7	Milky	1.2	+
	TUTVU21	IT-1263	II	1,000	IX	GCD	5–7	Translucent	1.1	+
	TUTVU22	IT-1263	II	1,000	IX	CCD	5–7	Translucent	1.1	+
	TUTVU23	IT-1263	II	1,000	IX	CCD	5–7	Translucent	1.1	+
	TUTVU24	IT-1263	II	1,000	IX	CCD	5–7	Translucent	1.1	+
	TUTVU25	IT-18	XVI	1,000	IX	CCD	5–7	Milky	1.1	+
	TUTVU26	IT-18	XVI	1,000	IX	CCD	5–7	Milky	1.1	+
	TUTVU27	IT-18	XVI	1,000	IX	CCD	5–7	Milky	1.0	+
	TUTVU28	IT-18	XVI	1,000	IX	CCD	5–7	White	1.0	+
	TUTVU-1	IT-18	VIII	—	—	—	7–10	Milky	0.7	−
	TUTVU-2	IT-18	XVI	—	—	—	7–10	Milky	1.8	−
	TUTVU-3	IT-18	XV	—	—	—	5–7	Milky	1.8	−
	TUTVU-4	IT-1263	SA	—	—	—	5–7	Milky	1.7	−
	TUTVU-5	IT-1263	SA	—	—	—	5–7	Milky	1.7	−
	TUTVU-6	IT-1263	SA	—	—	—	5–7	Milky	1.7	−
	TUTVU-7	IT-1263	XVII	—	—	—	5–7	Milky	1.6	−
	TUTVU-8	IT-1263	SA	—	—	—	5–7	Translucent	1.7	−
	TUTVU-9	IT-1263	XVII	—	—	—	5–7	Translucent	1.7	−
	TUTVU-10	IT-18	VIII	—	—	—	5–7	Translucent	1.6	−
	TUTVU-11	IT-1263	XVI	—	—	—	5–7	Translucent	1.8	−
	TUTVU-12	IT-18	XVI	—	—	—	5–7	Translucent	1.5	−
Ruace	TUTVU29	IT-1263	VII	500	II	IDF	5–7	Translucent	1.0	+
	TUTVU30	IT-1263	XVI	750	V	JGG	7–10	Milky	1.0	+
	TUTVU31	IT-1263	SA	730	IV	KHH	2–4	Translucent	2.3	+
	TUTVU32	IT-18	IX	1.000	IX	LED	7–10	Milky	1.0	+
	TUTVU33	IT-18	I	258	I	MII	2–4	White	2.1	+
	TUTVU34	IT-18	X	1,000	IX	AED	5–7	Milky	1.1	+
	TUTVU35	IT-1263	XII	1,000	IX	GCD	5–7	White	1.2	+
	TUTVU36	IT-1263	XII	1,000	IX	GCD	5–7	Milky	1.1	+
	TUTVU37	IT-1263	IX	NA	—	—	7–10	Milky	1.0	+
	TUTVU38	IT-1263	XIII	1,200	XI	NJD	5–7	Milky	1.1	+
	TUTVU39	IT-1263	XI	1,000	IX	OCD	5–7	Milky	1.1	+
	TUTVU40	IT-1263	XIII	500	II	DDJ	2–4	Milky	2.5	+
	TUTVU41	IT-1263	XII	980	VIII	GCD	7–10	White	1.1	+
	TUTVU42	IT-18	I	770	VI	PKD	2–4	White	2.4	+
	TUTVU43	IT-18	XII	550	III	QL-	5–7	White	1.1	+
	TUTVU44	IT-18	XVII	980	VIII	EAD	5–7	White	0.8	+
	TUTVU45	IT-1263	XVII	980	VIII	EAD	7–10	Translucent	0.8	+
	TUTVU46	IT-1263	X	980	VIII	CCD	7–10	Translucent	0.9	+
	TUTVU47	IT-18	I	550	III	QKK	7–10	Milky	0.7	+
	TUTVU48	IT-18	III	980	VIII	CCD	7–10	White	0.7	+
	TUTVU49	IT-18	III	NA	—	—	7–10	Translucent	1.0	+
	TUTVU50	IT-18	XII	1,350	XII	QM-	2–4	Translucent	2.1	+
	TUTVU51	IT-18	XV	1,000	IX	CCD	5–7	Milky	1.0	+
	TUTVU52	IT-1263	XV	1,000	IX	CCD	5–7	Milky	1.0	+
	TUTVU53	IT-1263	VI	1,000	IX	EAD	5–7	Milky	1.0	+
	TUTVU54	IT-1263	VI	500, 258	XIII	RNL	5–7	Milky	0.7	+
	TUTVU55	IT-1263	III	1,000	IX	CCD	5–7	White	0.8	+
	TUTVU56	IT-1263	III	1,000	IX	CCD	5–7	Translucent	0.9	+
	TUTVU57	IT-1263	III	1,000	IX	CCD	5–7	Translucent	0.8	+
	TUTVU58	IT-18	XII	600, 500	XIV	DOE	5–7	Translucent	1.1	+
	TUTVU59	IT-18	II	500	II	IDM	5–7	Translucent	1.1	+
	TUTVU60	IT-18	III	1,000	IX	CAD	7–10	White	1.0	+
	TUTVU101	IT-18	IX	—	—	—	5–7	Milky	1.8	−
	TUTVU102	IT-18	I	—	—	—	5–7	White	1.9	−
	TUTVU103	IT-18	XIV	—	—	—	5–7	White	1.5	−
	TUTVU104	IT-18	XIV	—	—	—	5–7	White	1.4	−
	TUTVU105	IT-18	X	—	—	—	5–7	Milky	1.7	−
	TUTVU106	IT-18	X	—	—	—	5–7	Milky	1.3	−
	TUTVU107	IT1263	XVII	—	—	—	5–7	Milky	1.4	−
	TUTVU108	IT-18	XIII	—	—	—	7–10	White	1.0	−
	TUTVU109	IT-18	VI	—	—	—	7–10	White	1.8	−
	TUTVU110	IT-18	X	—	—	—	7–10	Milky	1.2	−
Sussundenga	TUTVU61	IT-18	III	1,000	IX	CAD	5–7	White	1.0	+
	TUTVU62	IT-1263	III	1,000	IX	CAD	5–7	White	0.9	+
	TUTVU63	IT-18	SA	1,000	IX	CAD	5–7	Milky	1.0	+
	TUTVU64	IT-18	XIV	1,000	IX	CAD	5–7	Translucent	0.8	+
	TUTVU65	IT-18	VI	1,000, 258	XV	AAN	7–10	Translucent	0.9	+
	TUTVU66	IT-1263	XIII	1,000, 500	XVI	SAO	7–10	White	1.1	+
	TUTVU67	IT-1263	VII	1,200	XI	TAP	2–4	Translucent	2.4	+
	TUTVU68	IT-18	I	1,200	XI	KPQ	2–4	Translucent	2.3	+
	TUTVU69	IT-18	I	1,000	IX	CAD	7–10	White	1.0	+
	TUTVU70	IT-1263	III	1,000	IX	CAD	7–10	Milky	1.0	+
	TUTVU71	IT-18	II	1,000	IX	UAQ	7–10	Milky	1.0	+
	TUTVU72	IT-1263	III	1,000	IX	CAD	7–10	Milky	1.1	+
	TUTVU73	IT-1263	III	1,000	IX	CAD	7–10	White	1.0	+
	TUTVU74	IT-18	III	1,000	IX	KAD	7–10	Translucent	0.8	+
	TUTVU75	IT-18	VIII	1,000	IX	CAD	7–10	Translucent	0.8	+
	TUTVU76	IT-18	III	1,000	IX	CAD	5–7	Milky	0.8	+
	TUTVU77	IT-1263	XIV	800	VII	UKQ	5–7	Milky	0.9	+
	TUTVU78	IT-1263	XIV	1,000	IX	CAD	5–7	White	0.9	+
	TUTVU79	IT-18	XIV	1,000	IX	CAD	5–7	Milky	0.8	+
	TUTVU80	IT-1263	XVI	1,000	IX	CAD	5–7	White	1.1	+
	TUTVU81	IT-1263	XI	1,000	IX	CAD	5–7	Milky	1.1	+
	TUTVU82	IT-18	XI	1,000	IX	CAD	5–7	Milky	1.1	+
	TUTVU83	IT-1263	XVI	1,000	IX	CAD	5–7	Milky	1.1	+
	TUTVU84	IT-18	X	1,000	IX	CAD	5–7	Milky	1.0	+
	TUTVU85	IT-1263	X	1,000	IX	CAD	5–7	Milky	1.0	+
	TUTVU86	IT-1263	X	1,000	IX	CA-	5–7	Milky	0.9	+
	TUTVU87	IT-1263	X	1,000	IX	CAD	5–7	White	0.9	+
	TUTVU88	IT-18	X	1,000	IX	CAD	5–7	White	0.9	+
	TUTVU89	IT-1263	X	1,000	IX	CAD	5–7	White	0.8	+
	TUTVU90	IT-18	X	1,000	IX	CAD	5–7	Milky	0.8	+
	TUTVU91	IT-18	X	1,000	IX	CAD	5–7	Milky	0.8	+
	TUTVU92	IT-1263	X	500	II	CCR	5–7	Milky	1.1	+
	TUTVU93	IT-18	XIII	1,000	IX	CAD	5–7	Milky	1.0	+
	TUTVU94	IT-18	III	1,000	IX	CAD	5–7	Translucent	1.1	+
	TUTVU95	IT-18	V	1,000	IX	CAD	5–7	Translucent	1.0	+
	TUTVU96	IT-18	III	1,000	IX	CAD	5–7	Translucent	1.0	+
	TUTVU97	IT-1263	III	1,000	IX	CAD	5–7	Translucent	1.0	+
	TUTVU98	IT-18	III	600, 500	XIV	DQS	5–7	Translucent	0.8	+
	TUTVU99	IT-1263	V	1,000, 500	XVI	VRT	5–7	Milky	0.9	+
	TUTVU100	IT-1263	V	—	—	—	5–7	White	1.9	−
	TUTVU112	IT-1263	IV	—	—	—	7–10	Milky	1.4	−
Inoculant	BIOFIX			1,000, 1,200	XVII	WSU				

a NA, not amplified. —, not determined.

The BOX-PCR clustering data were used to analyze the diversity indices of the bacterial isolates from the three study sites. The types of bacterial isolates varied across sites (Fig. 1). The Shannon diversity index (*H*′) was highest (1.12) at Muriaze and lowest (0.09) at Sussundenga. The values for the Margalef index (R_1_), which is a measure of species richness, were similar across the three experimental sites, at 7.55, 8.64, and 8.07 for Muriaze, Ruace, and Sussundenga, respectively. The values for the Pielou index (E_1_), which measures species evenness, were similar at the Muriaze and Ruace experimental sites (1.01 and 0.92) but lowest (0.08) at Sussundenga.

Ninety-nine out of the 122 isolates (81%) effectively induced nodule formation in cowpea variety IT-18, the homologous host. Of this number, 28%, 32%, and 39% originated from Muriaze, Ruace, and Sussundenga, respectively.

The 99 effective rhizobial isolates differed significantly in morphological characteristics, with 94% of the isolates forming visible colonies on yeast mannitol agar (YMA) plates in 5 to10 days and only 6% forming visible colonies in 2 to 4 days. About 50% of the colonies were milky, 25% translucent, 37% gummy, and 21% secreting exopolysaccharide. Colony size varied from 0.8 to 2.4 mm in diameter.

### PCR-RFLP analysis of the 16S-23S rRNA genomic region.

The PCR-amplified products of the 16S-23S rRNA ITS region of rhizobial DNA showed single polymorphic bands, ranging from 258 to 1,350 bp in size, except for isolates TUTVU17, TUTVU54, TUTVU58, TUTVU65, TUTVU66, TUTVU98, and TUTVU99, which produced two fragments each (Table 1). The commercial inoculant strain (BIOFIX) also produced double bands and was distinct from the test isolates. Based on the differences in band size, the rhizobial isolates were grouped into 17 distinctive ITS types, numbered I to XVII (Table 1). The majority of rhizobial isolates from the three experimental sites grouped within ITS type IX with a band size of 1,000 bp (Table 1). Rhizobial isolates TUTVU37 and TUTVU49, however, failed to amplify.

Gel electrophoresis of products from the restriction endonuclease-digested PCR-amplified ITS genomic region indicated the presence of different enzyme recognition sites, as distinct polymorphic bands were observed. From the binary scoring, the HaeII restriction endonuclease enzyme was the most discriminatory, yielding 23 distinct fingerprint profiles (A to W), while the HindIII and HinfI restriction endonuclease enzymes yielded 19 (A to S) and 21 (A to U) distinct fingerprint profiles, respectively (Table 1).

**FIG 2:**
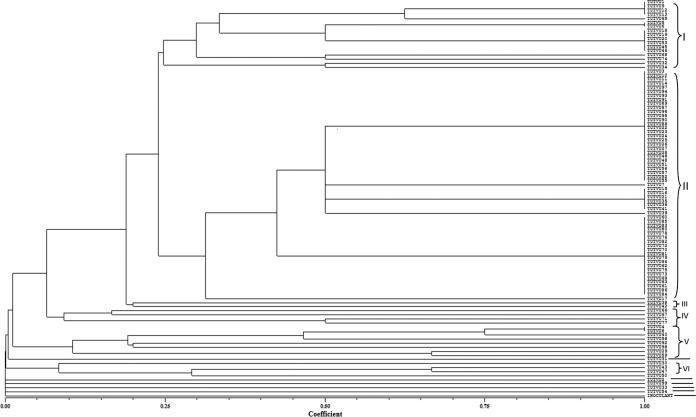
Dendrogram generated from ITS-RFLP banding pattern of rhizobial isolates nodulating cowpea in Mozambique.

The combined ITS-RFLP cluster analysis revealed six distinct ITS-RFLP clusters (I to VI) at 50% Jaccard's similarity coefficient (Fig. 2). Five of the rhizobial isolates (namely, TUTVU31, TUTVU2, TUTVU99, TUTVU33, and TUTVU54) were very distinct, as they showed no similarity with the other test isolates at 0 to 0.1% Jaccard's similarity coefficient. The commercial inoculant (BIOFIX) was highly distinct, at 0% Jaccard's similarity coefficient. Clade II had 57% of the rhizobial isolates, of which 51% originated from Sussundenga site. Seven groups of rhizobial isolates within clades I, II, and V, which comprised between 2 and 28 rhizobial isolates, each showed 100% Jaccard's similarity coefficient. Isolates from all the three study sites were represented in four of the seven groups. Clades III and VI each comprised two and four test isolates from the Ruace site only, while clade IV comprised four test isolates exclusively from Sussundenga. Based on cluster analysis, most of the isolates from the different experimental sites showed a high level of similarity at the ITS genomic level.

Representative isolates from each cluster of the ITS-RFLP dendrogram were selected for further phylogenetic study. Following PCR amplification, full-length amplicons (1.5 kb) of the 16S rRNA gene were obtained for all 34 test rhizobial isolates. However, 11 gene sequences of poor quality were excluded from the 16S rRNA study. The BLAST_n_ sequence analysis of 23 test rhizobial isolates showed that 74% of the isolates had very high sequence similarities to Bradyrhizobium, while 26% had high gene sequence similarities with Rhizobium type strains. The phylogenetic analysis was therefore computed separately for each group of isolates (i.e., Bradyrhizobium and Rhizobium).

### The Bradyrhizobium lineage.

**FIG 3:**
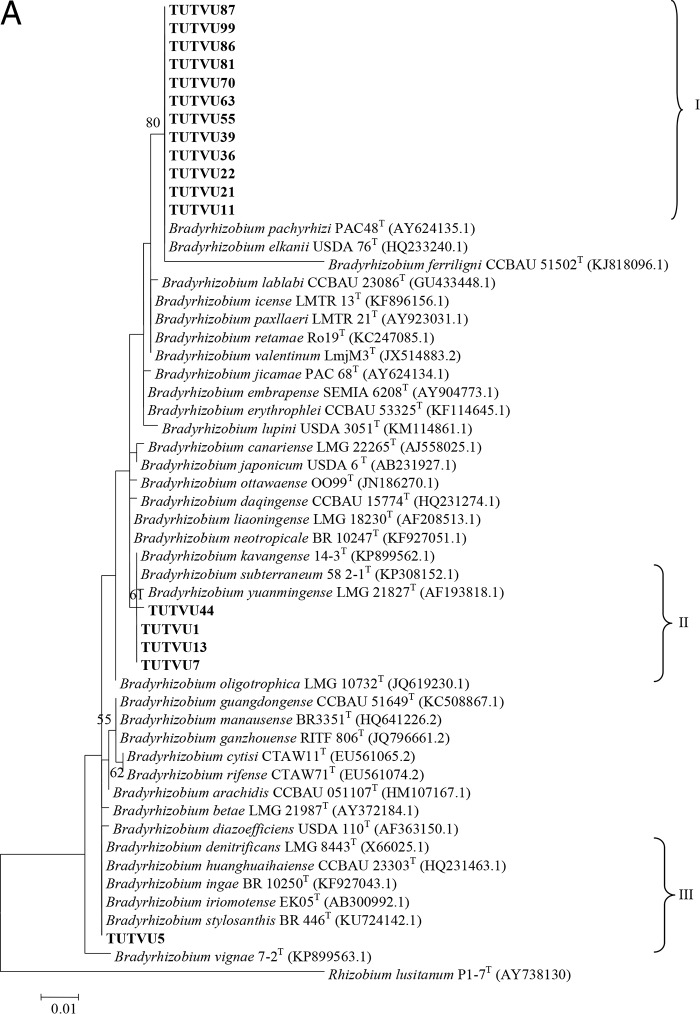

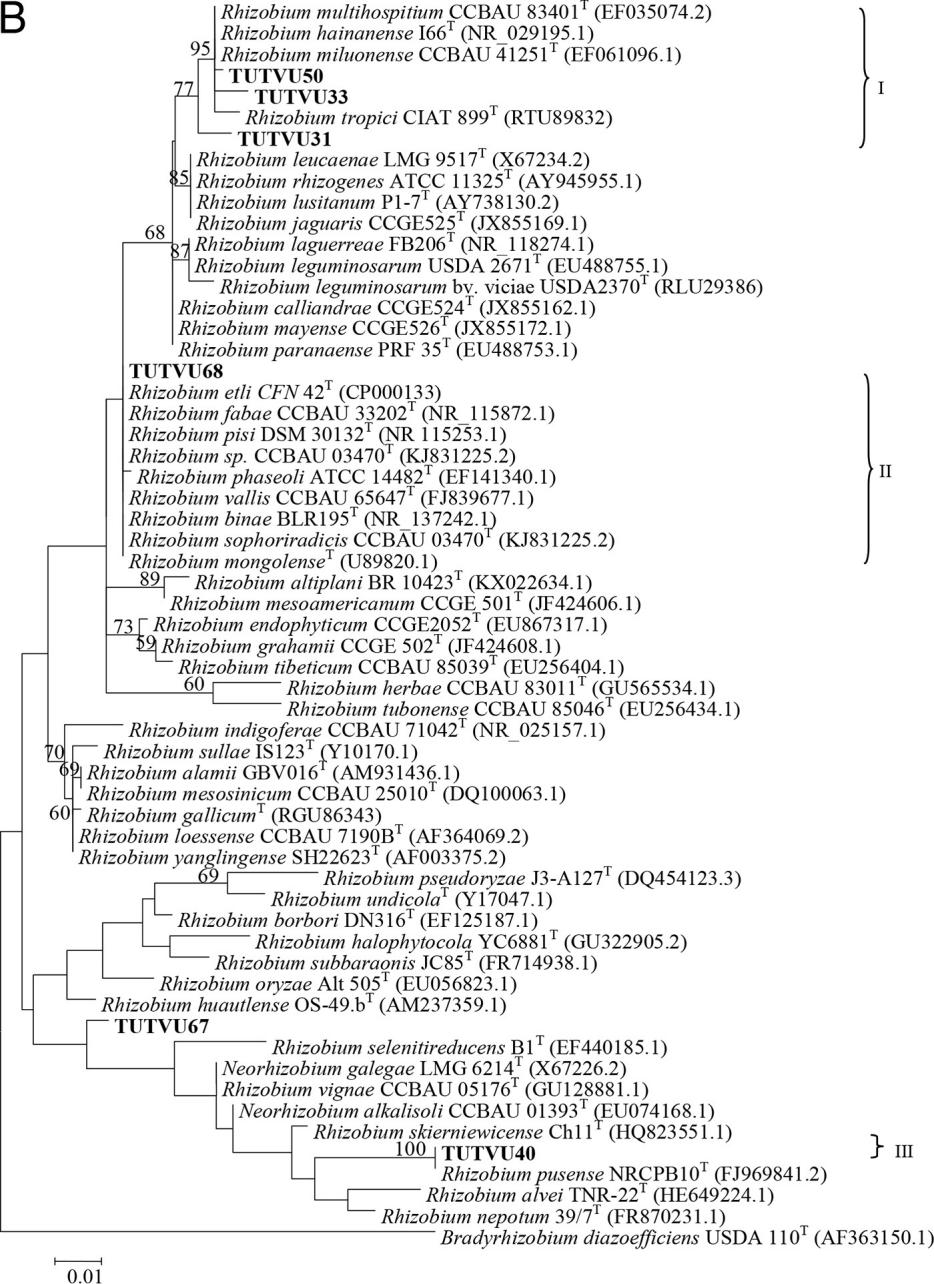
(A) Maximum likelihood based phylogenetic relationships between test isolates of cowpea root nodules and reference Bradyrhizobium type strains (NCBI) based on 16S rRNA gene sequences. Bootstrap values (1,000 replicates) of ≥50% are indicated at the nodes. (B) Maximum likelihood-based phylogenetic relationships between test isolates of cowpea root nodules and Rhizobium reference type strains (NCBI) based on 16S rRNA partial gene sequences. Bootstrap values are indicated at the nodes.

The 16S rRNA consensus sequence (534 bp) comprised 449 conserved, 85 variable, 14 parsimony informative, and 71 singleton sites (see Table S2 in the supplemental material). The maximum likelihood phylogeny of the 16S rRNA genes placed the test isolates in three distinct clades (I to III) (Fig. 3A). In clade I, isolates TUTVU87, TUTVU99, TUTVU86, TUTVU81, TUTVU70, TUTVU63, TUTVU55, TUTVU39, TUTVU36, TUTVU22, TUTVU21, and TUTVU11 grouped with the reference type strains of the B. elkanii group (i.e., B. elkanii and B. pachyrhizi) and showed 100% identical sequences, with 80% bootstrap support. Clade II comprised isolates TUTVU44, TUTVU1, TUTVU13, and TUTVU7 with 61% bootstrap support, and all of them had sequences identical to those of the type strains of B. kavangense and B. subterraneum, except for isolate TUTVU44, with 99.8% sequence identity. Isolate TUTVU5 shared identical sequences with the reference type strains of B. denitrificans, B. huanghuaihaisense, B. ingae, B. iriomotense, and B. stylosanthis in clade III, with 50% bootstrap support.

### The Rhizobium lineage.

The 569-bp length of 16S rRNA consensus sequence contained 457 conserved, 112 variable, 72 parsimony, and 40 singleton sites (Table S2). The 16S rRNA maximum likelihood phylogeny placed the test isolates in three (I to III) distinct clades (Fig. 3B). In clade I, isolates TUTVU50 and TUTVU33 grouped together with the type strains of R. hainanense, R. multihospitium, and R. miluonense, with 95% bootstrap support and 99.2 to 99.8% sequence identity, while isolate TUTVU31 formed an outgroup of clade I. Clade II comprised isolate TUTVU68 and the type strains of *R. etli*, R. fabae, R. pisi, R. binae, R. sophoriradicis, R. mongolense, and R. vallis, with 100% sequence identity. In clade III, isolate TUTVU40 had a sequence identical to that of the type strain of R. pusense, with 100% bootstrap support, while isolate TUTVU67 formed an outgroup of clade III.

### Sequence and phylogenetic analysis of housekeeping genes.

The PCR-amplified products of the *gyrB*, *rpoB*, *glnII*, and *recA* housekeeping genes yielded single fragments of approximately 750, 500, 650, and 700 bp, respectively. As done for the 16S rRNA phylogenetic analysis, the sequences of housekeeping genes were compared with published type reference strain sequences using the BLAST_n_ program of the NCBI (GenBank). These genes had high sequence similarities to the reference type strains of two distinct rhizobial genera (Bradyrhizobium and Rhizobium). The phylogenetic analyses were again done separately for the two groups of rhizobial strains (i.e., Bradyrhizobium and Rhizobium).

### Bradyrhizobium lineage gene sequence analysis.

Due to the poor quality of some sequences obtained, only 24 *glnII*, 25 *gyrB*, 21 *rpoB*, and 26 *recA* housekeeping gene sequences of the test isolates were subjected to single-gene *glnII* (244 bp), *gyrB* (358 bp), *rpoB* (226 bp), and *recA* (301 bp) phylogenetic analysis. The *gyrB* housekeeping gene had the highest number (112) of parsimony informative sites, while the *glnII* housekeeping gene had the lowest number (18) of singleton sites (Table S2).

The maximum likelihood phylogenies were generally congruent for three (*glnII*, *gyrB*, and *rpoB*) of the four housekeeping single-gene phylogenies, although there were some slight variations in the sequence identity of a few test isolates. In each tree topology, the test isolates grouped into four (I to IV) distinct clades (Fig. S1a, S2a, and S3a), with most of them clustering within similar clades across all three housekeeping genes. For example, clade I consisted of isolates TUTVU3, TUTVU11, TUTVU14, TUTVU22, TUTRU30, TUTVU36, TUTVU39, TUTVU47, TUTVU55, TUTVU59, TUTVU63, TUTVU66, TUTVU70, TUTVU77, TUTVU81, TUTVU86, TUTVU87, TUTVU92, and TUTVU99 in the four housekeeping gene phylogenies and had between 97.1 and 100% sequence identity to the type reference strains of B. pachyrhizi, B. elkanii, B. viridifuturi, and B. ferriligni. The only exception was isolate TUTVU66, which grouped with the type strain of B. arachidis, with 95% bootstrap support and 99.1% sequence identity in clade III of the *glnII* phylogeny, and isolate TUTVU21, which had a sequence identical to that of the type strain of B. elkanii in the *rpoB* gene but had a proximal relationship to the type strains of B. embrapense and B. viridifuturi, with 95.4% sequence identity and 57% bootstrap support in the *glnII* gene phylogeny.

In a similar manner, clade II, which comprised isolates TUTVU13, inoculant strain, and TUTVU44, showed a proximal relationship with the type strain of *B*. diazoefficiens, with 96.7 to 100% sequence identity in the *gyrB* and *glnII* phylogenies. But in the *rpoB* phylogeny, clade II comprised isolates TUTVU13, TUTVU1, and TUTVU44 together with the type strain of B. yuanmingense and shared 82% bootstrap support and 98.2 to 100% sequence identity. Isolate TUTVU1, however, showed discordance in the *glnII* phylogram. Isolates TUTVU54, TUTVU7, and TUTVU5 formed outgroups of clade II in the *glnII* phylogeny but not in the *rpoB* phylogeny, while isolate TUTVU5 grouped in clade III with the type strain of B. arachidis. Isolates TUTVU54 and TUTVU7 also grouped in clade IV with the type strain of B. liaoningense, with 52% and 62% bootstrap support, respectively. In the *gyrB* phylogeny, isolate TUTVU5 had 96.6% sequence identity with the type strain of B. stylosanthis, with 64% bootstrap support. Isolates TUTVU14, TUTVU39, TUTVU55, TUTVU63, and TUTVU87 were consistently grouped with the type strain of B. pachyrhizi in all three housekeeping gene phylogenies.

A discrepancy was, however, observed in the *recA* phylogeny (Fig. S4). For example, in clade II, isolates TUTVU7, TUTVU44, TUTVU13, and TUTVU54 had a proximal relationship with the type strain of B. subterraneum, while it showed a proximal relationship with the type strain of *B*. diazoefficiens in the *gyrB* and *glnII* phylogenies. Similarly, in clade III, isolate TUTVU63 grouped with the type strain of B. stylosanthis in this gene but also consistently showed a nearly identical relationship with the type strain of *B*. pachyrhizi in the other housekeeping gene phylogenies.

### The Rhizobium lineage.

The phylogenetic analysis was performed using sequences individually aligned with ClustalW for the *glnII* (358 bp), *gyrB* (577 bp), and *rpoB* (218 bp) housekeeping genes along with reference type strains obtained from the NCBI GenBank database.

The maximum likelihood phylogenetic analyses were congruent for the *glnII*, *gyrB*, and *rpoB* housekeeping genes (Fig. S1b, S2b, and S3b). For example, isolates TUTVU50 and TUTVU31 showed high genetic relatedness to type strains in the R. tropici group, with high bootstrap support. However, isolates TUTVU33 and TUTVU67 also clustered with the type strain of R. galegae, with 100% bootstrap support in the *glnII* and *gyrB* phylogenies, while in the *rpoB* phylogeny, isolate TUTVU33 grouped with the type strain of R. mesosinicum, with 86% bootstrap support. In contrast, isolate TUTVU40 showed genetic relatedness to Neorhizobium pusense in all three housekeeping gene phylogenies.

**FIG 4:**
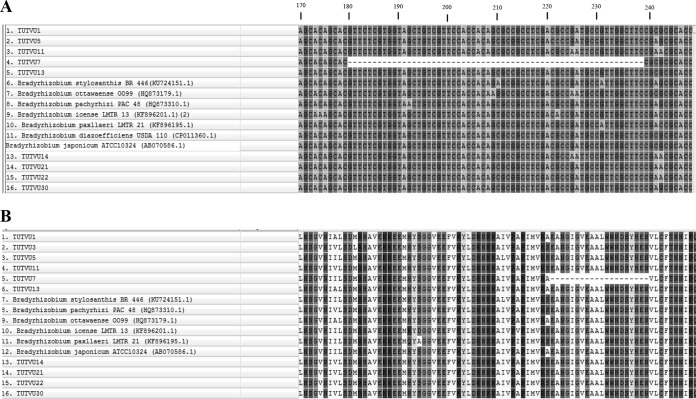
Sequence alignment of amplicons of *gyrB* gene of test cowpea isolates. (A) Nucleotide sequence alignment showing 60-bp deletion regions; (B) amino acid sequence alignment regions showing 20-amino-acid deletion regions.

Interestingly, the *gyrB* nucleotide sequence of isolate TUTVU7 showed variation (gaps) in comparison to test and type bradyrhizobial strains. The 60-bp natural deletion was observed in the *gyrB* genomic region of isolate TUTVU7 (Fig. 4A). The annotation result suggested the occurrence of a deletion in the coding region (Fig. 4B).

### Concatenated gene phylogenies.

A concatenated gene sequence analysis was performed to refine the phylogenetic positions of the test isolates within the Bradyrhizobium and Rhizobium lineages. A maximum likelihood phylogeny was constructed using concatenated aligned sequences of the test isolates and type reference strains common across the four housekeeping genes.

### The Bradyrhizobium lineage.

**FIG 5:**
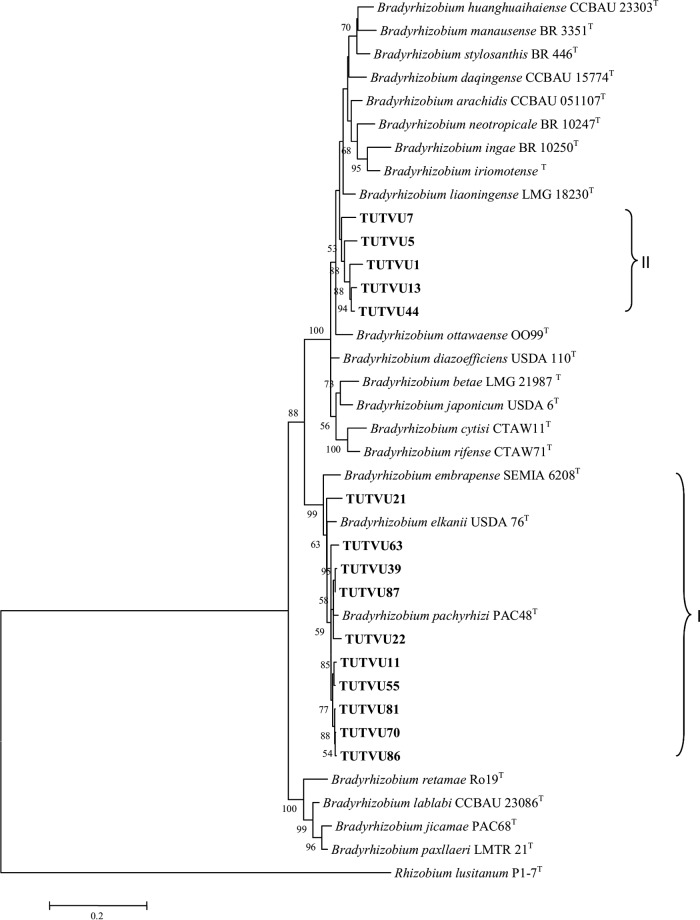
Maximum likelihood-based concatenated (*glnII* plus *gyrB* plus *recA* plus *rpoB*) phylogenetic tree of test Bradyrhizobium isolates and reference type sequences (GenBank). Bootstrap values are indicated at the nodes.

For the Bradyrhizobium lineage, 15 test isolates were included in the concatenated (*glnII* plus *gyrB* plus *recA* plus *rpoB*) gene sequence analysis. The consensus sequence of 1,128 bp comprised 648 conserved, 480 variable, 290 parsimony, and 190 singleton sites (Table S2). In the concatenated tree topology, the test isolates grouped into two distinct (I and II) clades (Fig. 5). Ten isolates (namely, TUTVU86, TUTVU21, TUTVU22, TUTVU70, TUTVU81, TUTVU11, TUTVU55, TUTVU39, TUTVU63, and TUTVU87) clustered in clade I, with 99% bootstrap support. In this clade, test isolates TUTVU21 and TUTVU22 had between 98.1 and 98.7% sequence identities with the type strain of B. pachyrhizi. In clade II, isolates TUTVU7, TUTVU5, TUTVU1, TUTVU13, and TUTVU44 grouped together without any type reference strains.

### The Rhizobium lineage.

**FIG 6:**
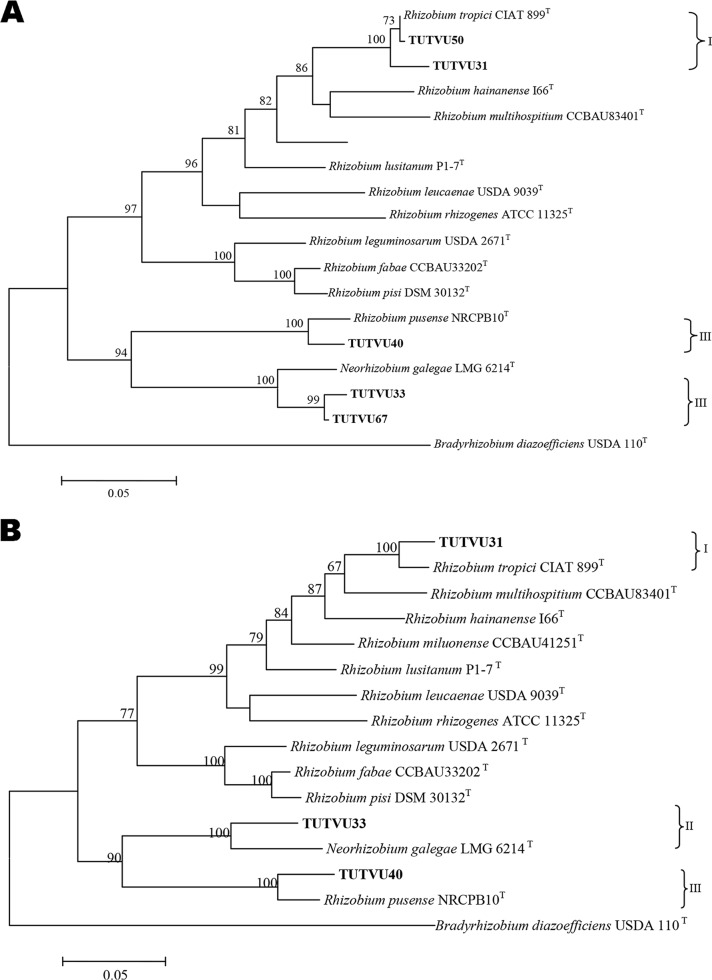
(A) Maximum likelihood-based concatenated (*glnII* plus *gyrB*) phylogenetic tree of test Rhizobium isolates and reference type sequences (GenBank). Bootstrap values are indicated at the nodes. (B) Maximum likelihood-based concatenated (*glnII* plus *gyrB* plus *rpoB*) phylogenetic tree of test Rhizobium isolates and reference type sequences (GenBank). Bootstrap values are indicated at the nodes.

For the Rhizobium lineage, concatenated housekeeping gene analysis was performed in two subsets (*glnII* plus *gyrB*) and (*glnII* plus *gyrB* plus *rpoB*) to account for inconsistencies in the number of test isolates in the individual phylogenetic trees (Fig. 6A and B). The consensus analysis of the *glnII* plus *gyrB* concatenated gene sequences of 976 bp consisted of 558 conserved and 418 variable sites, while the concatenated gene sequences (*glnII* plus *gyrB* plus *rpoB*) of 1,194 bp comprised 531 variable and 361 parsimony informative sites (Table S2).

In the maximum likelihood phylogeny of both concatenated housekeeping gene subsets, isolates TUTVU50 and TUTVU31 were closely related (99.6% and 97.7% sequence identity) to the type strain of R. tropici, while isolates TUTVU33 and TUTVU67 both showed low sequence identities (94.7% and 95.2%) with the type strain of R. galegae, with 100% bootstrap support (Fig. 6). Isolate TUTVU40 in clade III had a 96.8% sequence similarity with the type strain of Neorhizobium pusense, with 100% bootstrap support in both concatenated housekeeping gene subsets.

### Phylogenetic analysis of the *nifH* symbiotic gene sequences.

**FIG 7:**
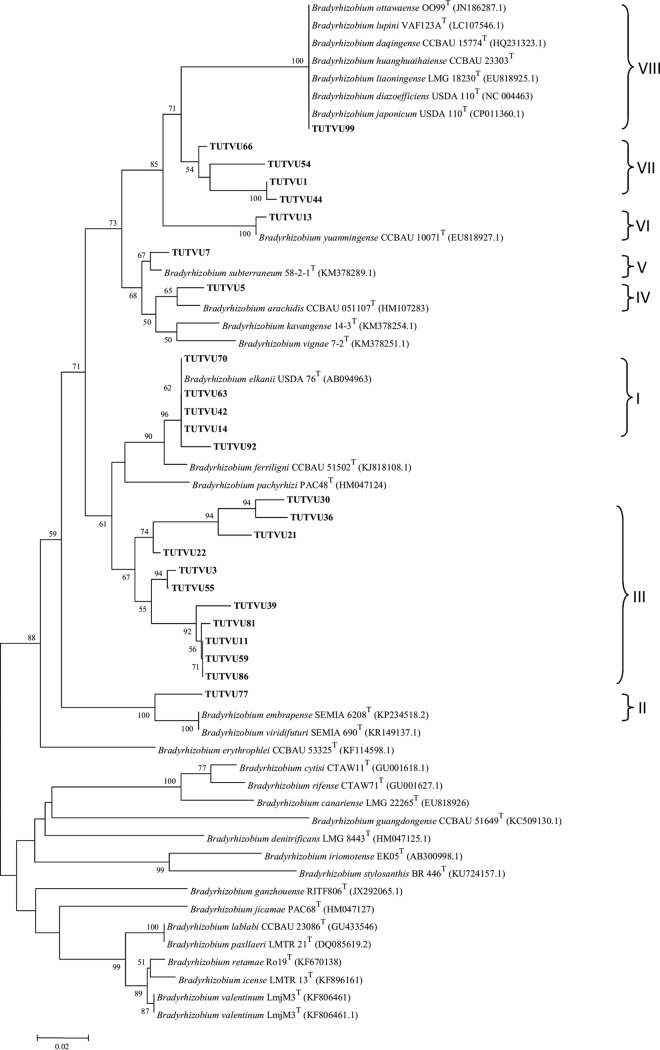
Maximum likelihood-based phylogenetic relationships between test isolates of cowpea root nodules and reference Bradyrhizobium type strains (GenBank) based on *nifH* gene sequences. Bootstrap values are indicated at the nodes.

The PCR-amplified products of the *nifH* gene yielded single polymorphic fragments 800 bp in length. In the maximum likelihood phylogeny, the test isolates grouped into eight distinct clades (I to VIII) (Fig. 7). Clades III and I comprised test isolates which previously clustered with the B. elkanii lineage in the housekeeping gene phylogenies. All the isolates in clade III, however, formed a monophyletic group, with no close relatedness to any known type strains. Clades II, IV, V, and VI each comprised isolates TUTVU77, TUTVU5, TUTVU7, and TUTVU13, which, respectively, had 99.5%, 97.9%, 99.1%, and 99.5% sequence similarities with the type strains of B. viridifuturi, B. arachidis, B. subterraneum, and B. yuanmingense. In clade VIII, isolate TUTVU99 had identical sequence similarity with reference type strains of B. japonicum, B. diazoefficiens, B. liaoningense, B. huanghuaihaiense, B. daqingense, B. ottawaense, and B. lupini, with 100% bootstrap support. However, test isolates TUTVU1, TUTVU44, TUTVU54, and TUTVU66 in clade VII grouped together but not with any known reference type strain.

### 16S rRNA metagenomic analysis.

A total of 127,013 reads were observed, and each sample recorded >10,000 reads. The observed reads were assigned to high taxonomic ranks. The analysis based on occurrence and relative abundance of different phyla suggested strong dissimilarities among bacterial communities in all the test rhizosphere soil samples. The results showed that at Sussundenga the rhizosphere of the two cowpea varieties attracted the highest number (18) of bacterial phyla, followed by Muriaze and then Ruace. We also found bacteria of unknown genera in the rhizosphere soils, and their numbers followed the same pattern as the identifiable phyla, in that these unidentified bacteria occurred in greater abundance in cowpea rhizosphere at Sussundenga (45.05 to 53.34%), followed by Muriaze and Ruace. However, of the betaproteobacteria found, the rhizosphere of IT-18 at Sussundenga and IT-1263 at Muriaze revealed the highest number of bradyrhizobial populations (data not shown).

## DISCUSSION

The benefits of legume N_2_ fixation can potentially be better exploited by exploring new biogeographic regions for novel rhizobia and legume germplasm, both of which can lead to the discovery of novel symbioses and elite microsymbionts in support of agriculture. Cowpea (Vigna unguiculata [L.] Walp.) is an important food legume in Mozambique, but its rhizobial diversity and phylogeny are still little understood.

In this study, the diversity of root nodule bacteria isolated from cowpea was assessed using PCR-based fingerprinting techniques. The BOXA1R subunit of the BOX repetitive elements in the bacterial genome is highly conserved; therefore, PCR amplification analysis of this region can be used to differentiate bacterial isolates (26). Although the region is highly conserved, genetic distances between repetitive sequences can vary in unrelated organisms (26). The technique has therefore been successfully employed in diversity studies of rhizobia to identify interstrain variations (27, 28).

The 122 cowpea root-nodule bacteria used in this study were grouped into 17 distinct clusters following analysis of their BOX-PCR-amplified products. However, within these 17 clades, there was 100% Jaccard's similarity coefficient for over half (68 out of 123) of the isolates, indicating a high level of relatedness among the isolates. The BOX-PCR results showed high genetic distance (0% Jaccard's similarity coefficient) in six isolates (namely, TUTVU31, TUTVU-5, TUTVU-4, TUTVU-6, TUTVU-8, and TUTVU63), suggesting that they are genetically distinct from the rest (0% coefficient).

The nodulation assay showed that 81% (99 out of 122) of the isolates could elicit nodule formation in cowpea (IT-18), in fulfilment of Koch's postulates (29). The 23 nonnodulating isolates are likely to be endophytes, as the presence of nodule endophytes has been reported for Vigna species (30). According to Peix et al. (9), endophytic bacteria can coexist with rhizobial strains in nodules without causing any visible harm, and sometimes they even play a supportive role in plant growth and N_2_ fixation. In fact, nodules may actually be an important ecological niche in the life cycle of these endophytes (31), since they are sheltered from environmental stress as well as competition for resources in the rhizosphere (32). In return, endophytic bacteria can support the growth of their host through secretion of metabolites such as indole-3-acetic acid (IAA) (33), lumicrome, and riboflavin (34).

The use of morphological traits in this study revealed considerable variations among the isolates. About 94% of them that were proven by Koch's postulates to be nodule-forming rhizobia took 5 to 10 days to grow on YMA plates, while 6% took 2 to 4 days. This could suggest the presence of fast-growing (Rhizobium) and slow-growing (Bradyrhizobium) rhizobial species among the isolates. Various studies have reported the ability of both Rhizobium and Bradyrhizobium to nodulate cowpea (35, 36). The results of this study are therefore consistent with those reports. The results of this study also further confirmed the nodulation promiscuity of cowpea as a host plant (37).

Multiple alleles in the rRNA operon of bacteria account for spacer variations between and among species and can be used for typing and identification of rhizobial species (38). The ITS ribosomal genes have been shown to be good markers for assessing rhizobial diversity (16). In this study, ITS-PCR analysis grouped the 99 rhizobial isolates into 17 distinct ITS types, which showed very high polymorphism (258 to 1,350 bp) within the ITS region of the rhizobial genomic DNA. These results have therefore confirmed the discriminatory power of ITS tools in rhizobial diversity studies (16, 39, 40). The differences in product size have been attributed to variations in the number, length, and composition of the ITS spacer regions of various bacteria (38), partly explained by the insertion and/or deletion of tRNA genes in the ITS regions (41). Furthermore, the nonamplification of the ITS regions of isolates TUTVU37 and TUTVU49 may be explained by the possibility of an absence of tRNA genes within their ITS regions and/or primer and thermal cycling incompatibility. It has been suggested that the presence of the double bands observed following gel electrophoresis of the ITS-PCR-amplified products in isolates TUTVU54, TUTVU58, TUTVU65, TUTVU66, TUTVU17, TUTVU98, and TUTVU99 could be due to variations in length and/or the formation of heteroduplex DNA structures and single-stranded DNA during PCR amplification (42, 43). However, this could also be attributed to base deletions in a single strand of the DNA duplex which resulted in differences in electrophoretic mobility (44).

The ITS-RFLP analysis delineates bacteria based on the differences in the recognition sites for restriction endonuclease enzymes within the ITS regions of the genomic DNA. This has been shown to be applicable to rhizobial DNA fingerprinting (16, 45, 46). In this study, ITS-RFLP analysis of 99 rhizobial isolates yielded between 19 and 23 different fingerprint profiles for each endonuclease enzyme, suggesting the presence of different recognition sites for the endonuclease enzymes. Five isolates (namely, TUTVU33, TUTVU42, TUTVU47, TUTVU50, and TUTVU62), however, appeared not to have the recognition sites (GCGC) within their ITS regions for the four base-cutting HaeII restriction endonuclease, as they failed to digest with this enzyme. This is similar to the findings of Ngo Nkot et al. (47).

In the combined ITS-RFLP cluster analysis, 100% Jaccard's similarity coefficient was observed in 68 out of the 99 rhizobial isolates located in different clusters, suggesting that despite the differences observed for each restriction endonuclease enzyme when analyzed singly, most of the isolates had a similar number of enzyme recognition sites for the three restriction endonuclease enzymes. This could be attributed to intraspecific differences between and among the isolates (47). However, unlike other studies, where a correlation was found between rhizobial diversity and either the land use system (47) or the environment (48), the rhizobial isolates in this study were distributed across all clusters irrespective of geographic origin or cowpea variety (Table 1). For example, in clade II, which contained more than half (57%) of the isolates, 28%, 21%, and 51% were, respectively, isolated from root nodules collected at Muriaze, Ruace, and Sussundenga. Similarly, 49% and 51% of microsymbionts were isolated from cowpea varieties IT-18 and IT-1263, respectively. However, there was a 100% Jaccard's similarity among the rhizobial isolates in clades I and II, thus suggesting the presence of similar recognition sites for all three endonuclease enzymes within the ITS genomic regions of these isolates. The highest heterogeneity was found among five rhizobial isolates (namely, TUTVU31, TUTVU2, TUTVU99, TUTVU33, and TUTVU54) which showed marked distinctiveness based on their endonuclease enzyme recognition sites. This distinctiveness was also observed in the commercial strain BIOFIX, which suggests that this rhizobial strain is very different from the resident rhizobial populations of the experimental sites.

In the BOX-PCR analysis, the BIOFIX inoculant genome showed 100% similarity with only one isolate (TUTVU26), which suggests that nodule occupancy by the inoculant strain was very low. This could be attributed to poor competitiveness of the introduced strain compared to the resident native cowpea rhizobia in the soil, or incompatibility between the inoculant strain and the cowpea varieties used.

Within Africa, where the crop is indigenous, studies of cowpea rhizobia have found nodulation of the legume by species belonging to the genus Bradyrhizobium in Senegal, Botswana, Ghana, South Africa, Angola, and Namibia (7, 36, 37, 48) and to the genus Rhizobium based on rhizobial growth rate (35). However, beyond the African continent, cowpea nodulation by species of Rhizobium, Sinorhizobium, Ralstonia, Achromobacter, and Microvirga has also been reported (15, 23, 25, 49–51). In this study, the evolutionary relationships of cowpea rhizobia originating from three agroecological zones of Mozambique were determined using sequence analysis of their ribosomal, symbiotic, and housekeeping genes in order to ascertain the microsymbionts nodulating cowpea in that country.

Nucleotide sequence analysis of the 16S rRNA gene is universally accepted as a way to determine the phylogenetic relationships of all microorganisms on Earth (9). In this study, phylogenetic analysis of the 16S rRNA gene revealed the nodulation of cowpea by diverse Rhizobium and Bradyrhizobium species in Mozambican soils.

Housekeeping genes have been extensively used in Bradyrhizobium phylogeny in order to properly delineate closely related species (52–54). In this study, the single-gene phylogenies were highly congruent between and among the four housekeeping genes (*gyrB*, *glnII*, *recA*, and *rpoB*). This congruence was, however, predominant in the test isolates that were closely related to the B. elkanii lineage, a finding consistent with the 16S rRNA phylogeny. Beyond this, however, the test isolates showed various degrees of incongruence in the four housekeeping gene phylogenies. For example, although isolate TUTVU1 was closely related to B. diazoefficiens in the *gyrB* phylogeny, it showed a close relationship with B. yuanmingense in both the *glnII* and *rpoB* phylogenies. Similarly, isolates TUTVU13 and TUTVU44, which were closely related to B. yuanmingense in the *rpoB* phylogeny, grouped with B. diazoefficiens in the *gyrB* and *glnII* phylogenies. Like this in *recA* phylogeny, isolate TUTVU63, which grouped with the type strain of B. stylosanthis, consistently showed a proximal relationship with B. pachyrhizi in the other housekeeping gene phylogenies. These inconsistencies with housekeeping gene phylogenies could be attributed to horizontal gene transfer, subsequent recombination events, and/or differences in the evolutionary history of the gene, (55–58).

Single housekeeping gene phylogenies may therefore not always reflect organismal phylogeny, as they can be sensitive to unequal evolutionary rates among taxa and nucleotide sites within a single gene (59) and could lack adequate phylogenetic information for the resolution of all relationships (60). Phylogenetic information is therefore usually deduced from more than one locus in constructing evolutionary trees (52, 60). In this study, concatenated phylogeny clearly grouped the test isolates into two different major clades, thus clarifying the discrepancies that were found between single-gene phylogenies. Within the B. elknaii lineage (clade I), all the test isolates revealed less than 1% sequence divergence with B. pachyrhizi. Recently, B. pachyrhizi was identified as a cowpea microsymbiont in Angola (36) and Spain (24), although it was originally reported as the microsymbiont nodulating yam bean, Pachyrhizus erosus (61). Likewise, the nodulation of cowpea by B. arachidis, B. elkanii, and B. yuanmingense has been previously reported (24, 62).

Isolates TUTVU1, TUTVU5, TUTVU7, TUTVU13, and TUTVU44 obtained from root nodules of cowpea varieties IT-18 and IT-1263 at Muriaze and Ruace grouped together without any type reference strains and could therefore belong to a novel bradyrhizobial species in Mozambican soils.

This study further found that along with Bradyrhizobium species, fast-growing isolates belonging to Rhizobium also nodulated cowpea in Mozambican soils, a finding consistent with reports from Zimbabwe (35), Brazil (49), and China (22). But Rhizobium isolates could also form effective root nodules on cowpea variety IT-18 in this study and had identical to nearly identical sequences with three different Rhizobium type strains. This is in contrast to the report by Gronemeyer et al. (36) that fast-growing bacteria isolated from cowpea nodules in Namibia failed to induce nodulation in cowpea.

In this study, single housekeeping gene phylogenies showed a high degree of congruence between the *gyrB* and *glnII* phylogenies for all the test isolates, although there was a small variation in sequence identity with reference type strains. In the concatenated (*gyrB* plus *glnII*) phylogeny, isolates TUTVU50 and TUTVU31 clustered with R. tropici, with 100% bootstrap support, while isolates TUTVU33 and TUTVU67 were closely related to Neorhizobium galegae. Interestingly, Neorhizobium galegae, which was originally isolated from Galega officinalis (63) and has a worldwide distribution, has also been reported to be a microsymbiont of another Vigna species, Vigna radiata (64). Although isolate TUTVU40 consistently clustered with R. pusense in both subsets of the concatenated gene phylogenies, this reference type strain (R. pusense) was originally isolated from the rhizosphere of Cicer arientinum (65) and has not been reported as a nodulating bacterial symbiont of cowpea.

The *gyrB* product amino acid sequence length variation between TUTVU7 and other test isolates as well as reference type bradyrhizobial strains suggests some variation in their protein structure which might have affected stability and function of the protein. In an earlier study, aberration in the *gyrB* gene caused resistance to the antibiotic novobiocin in Escherichia coli (66).

Maximum likelihood phylogeny of the symbiotic *nifH* gene showed inconsistency with other core housekeeping gene phylogenies (Fig. 7). For example, all the isolates in clade III formed a monophyletic group without any reference type strain in the *nifH* phylogeny but clustered with B. elkanii in the core housekeeping genes. This suggests that there might be differences in the evolutionary history of chromosomal and symbiotic genes. The inconsistency of *nifH* and housekeeping gene phylogenies could be due to interstrain gene transfer and/or recombination of *nifH* sequences. In fact, the phylogenies of symbiotic genes are reported to differ from those of the core housekeeping genes (55, 67), an argument that is strongly supported by the *nifH* and housekeeping gene phylogenies obtained in this study. The clustering of isolates in clade VII without any type reference strain in *nifH* phylogeny probably indicates that the *nifH* gene of this clade has an origin different from that of known bradyrhizobial strains.

Taken together, the results of this study have identified the microsymboints nodulating cowpea in Mozambique. From the literature, this is the pioneer report of nodule occupancy by Rhizobium species in Africa, which indicates a wider rhizobial species boundary of the crop in its center of origin. The possibility of greater rhizobial diversity, however, cannot be ruled out, as this study covered only one locality in 3 out of the 10 agroecological zones of Mozambique. This study has contributed to the global database on the distribution of rhizobial species and reported the presence of diverse Bradyrhizobium spp. (B. pachyrhizi, B. elkanii, and B. yuanmingense) and a novel Bradyrhizobium sp., as well as R. tropici, R. pusense, and Neorhizobium galegae, as nodulating microsymbionts of cowpea in Mozambique. Given the large diversity of rhizobia nodulating cowpea in Mozambique, it could prove useful to evaluate these test isolates for symbiotic efficiency and competitiveness as a first step toward identifying superfixers for inoculant production in order to increase food and nutritional security. Furthermore, isolate TUTVU7 produced novel sequences of unknown origin, and this needs functional confirmation using gyrase DNA (*gyrB*) during replication and transcription of biological processes.

## MATERIALS AND METHODS

### Description of experimental sites.

**FIG 8:**
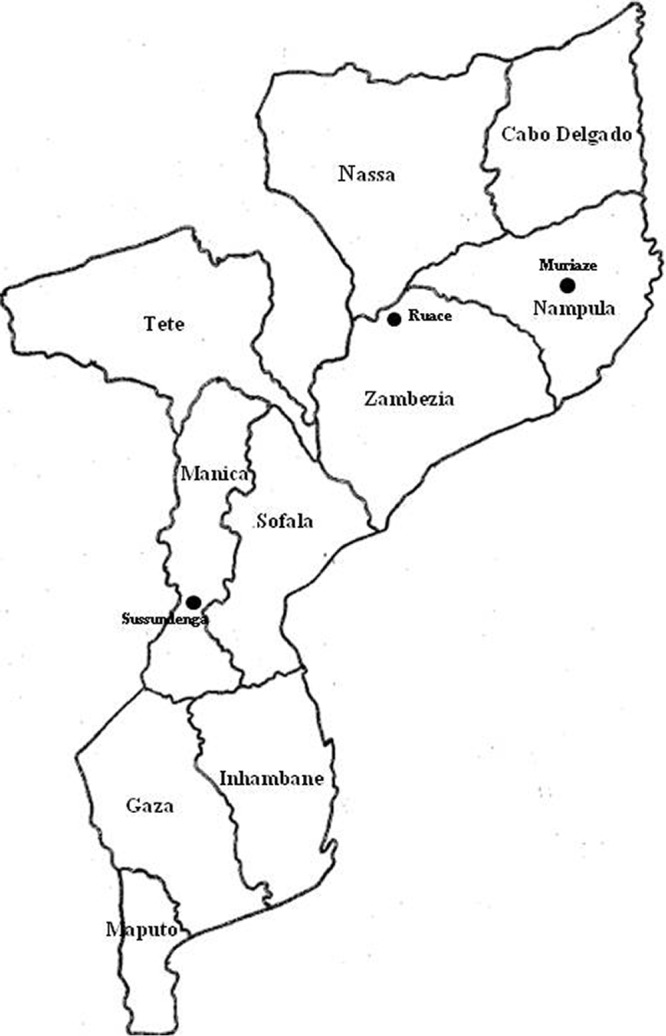
Three test locations in different provinces in Mozambique.

Two farmer-adopted improved cowpea varieties were used. These include IT-1263, which is semierect, with indeterminate growth, drought tolerant, and high yielding and produces dark brown large seeds, and IT-18, which is erect, with determinate growth, and high yielding and produces small light brown seeds. The field experiments were set up at three sites during the 2014-2015 cropping seasons: at Muriaze in Nampula Province of northern Mozambique, Ruace in Zambezia Province of central Mozambique, and Sussundenga in Manica Province of central Mozambique (Fig. 8).

The three sites are located in different agroecological zones (AEZs). Muriaze, in Nampula Province, is in AEZ 7, which is a vast region in northern Mozambique (68) with elevations ranging from 200 to 1,000 m above sea level and annual rainfall of 1,000 to 1,400 mm. Its soil types vary, with utisols and oxisols as the most predominant. Ruace, in Zambezia Province, is in AEZ 10, a high-elevation region, situated 1,000 m above sea level. The annual rainfall exceeds 1,200 mm, and the soils have a predominance of high clay utisols. Sussundenga, in Manica Province, is in AEZ 4, which lies 200 to 1,000 m above sea level. It has an annual rainfall of 1,000 to 1,200 mm and soils dominated by oxisols. The geographical and soil details of test locations are provided in Table 2.

**TABLE 2:** Geographical and soil information for test locations

Parameter	Muriaze	Ruace	Sussundenga
Environmental conditions			
Latitude	15^o^09′12.9″S	15^o^14′17.5″S	19^o^19′2.2″S
Longitude	39^o^19′20″E	36^o^43′44.8″E	33^o^14′22.5″E
Vegetation	Semiarid savannah	Grassland	Wooded grassland
Soil type	Sand-clay-loam	Clay-loam	Sand-loam
Elevation (m)	398	707	630
Temp (°C)	18.3–35.7	11.6–33.8	15.2–34.2
Cropping history	Fallow	Sesame	Maize
pH	6.38	5.93	6.41
Soil conditions			
Electrical conductivity (salts) (dS/m)	0.04	0.07	0.04
Organic P (mg · kg^−1^)	7.59	26.1	10.19
N (%)	0.12	0.05	0.09
K (mg · kg^−1^)	156.5	221	108
Ca (mg · kg^−1^)	1,009	803.5	408
Mg (mg · kg^−1^)	107.1	112.8	76.3
Na (mg · kg^−1^)	19.2	13.2	17.6
Cation exchange capacity [cmol(+)/kg]	7.5	7.2	3.5

Two rhizobial treatments were used (inoculated and uninoculated) for the two test varieties at all locations. All seeds were preinoculated in the shade with a commercial cowpea inoculant (BIOFIX; MEA, Kenya), according to the manufacturer's instructions.

### Root nodule sampling and isolation of bacteria.

Dark green healthy plants were sampled for nodules at 50% flowering to early podding stage. The root nodules were carefully detached and stored in vials containing silica gel at 4°C prior to molecular analysis. The nodules were surface sterilized using standard methods (29, 69). First, the nodules were rehydrated by soaking in sterile distilled water for a few hours and then placed in 95% ethanol for 10 s to break their surface tension. The nodules were surface sterilized in 3% (vol/vol) sodium hypochlorite for 4 min, rinsed in six changes of sterile distilled water, and transferred aseptically into sterile petri dishes for bacterial isolation. A loopful of last washed water was streaked on a yeast mannitol agar (YMA) plate to see if the nodule surface was well sterilized. Each sterilized root nodule was crushed aseptically in a drop of sterile distilled water, using sterile blunt forceps. A loopful of the macerate was streaked on YMA plates. The plates were incubated at 28°C and observed for bacterial growth up to 14 days. Visible colonies were purified by reculturing on YMA plates until single homogenous colonies were obtained. Based on their colony appearance on YMA, isolates were grouped as slow (>5 days) and fast (<4 days) growers. In addition, colony characteristics such as shape, color, texture, and size were scored for each rhizobial isolate (70).

Pure single colonies were maintained as working cultures on YMA slants in sterile McCartney bottles at 4°C, and another batch was stored at −20°C in 40% glycerol as stock culture.

### Nodulation assay.

An authentication assay was done for nodule formation on the homologous host by each bacterial isolate. For this, a loopful of each pure single colony isolate was cultured in 6 ml of YM broth to exponential growth phase (≈1 × 10^7^ to 1 × 10^8^ cells · ml^−1^) and used as inoculum. Seedlings of cowpea variety IT-18 were raised aseptically as described by Somasegaran and Hoben (69) and Woomer et al. (70) using beach sand as growth medium. The beach sand was washed, placed in 1-liter pots, covered with cotton and aluminum foil, and autoclaved at 121°C for 20 min. The experiment was set up in triplicates, and three seeds were sown per pot. The seedlings were thinned down to one plant per pot 3 days after germination. The bacterial inoculation used 2 ml of broth culture per seedling and was done aseptically under a laminar-flow hood 1 day after thinning. Uninoculated seedlings were included as negative controls, while 0.5 mM KNO_3_-fed seedlings served as positive controls. The pots were arranged in a randomized complete block design in the greenhouse. Watering of plants with sterile distilled water was done when necessary and was alternated with the supply of Broughton & Dilworth (71) nitrogen-free nutrient solution. Plant harvesting was done 3 weeks after inoculation for nodulation assessment.

### Total genomic DNA extraction and BOX-PCR fingerprinting.

Total genomic DNA was extracted using a GenElute bacterial DNA isolation kit according to the manufacturer's instructions (Sigma-Aldrich, USA). The integrity of the extracted DNA was evaluated on a 1% agarose gel stained with ethidium bromide. The genomic DNA was amplified using BOXA1R primer (26) to generate fingerprint patterns. DNA amplification was performed in a final reaction volume of 25 μl comprising 1 μl (50 to 80 ng) of genomic DNA, 3 μl (5×) of buffer, 1 μl (10 pM) of primer, 0.1 μl (5 U) of *Taq* polymerase (Bioline, USA), and 18.9 μl of sterile distilled water. The reaction mixture was incubated in a thermal cycler (T100; Bio-Rad, USA) at standard temperature profiles (Table 3). The PCR-amplified products (25 μl) were mixed with 5 μl (6×) of loading dye and placed in wells of 1.2% (wt/vol) agarose gel containing 1× Tris-acetate-EDTA (TAE) stained with ethidium bromide for gel electrophoresis at 95 V for 3 h. Gel imaging and documentation were done using a GEL Doc XR+ molecular imager (Bio-Rad). The data were analyzed using Bio-Numerics software (temporary license from Applied Maths, Belgium; permission received to publish). Similarity matrices were calculated using Jaccard's coefficient (72), and cluster analyses were generated using the unweighted pair group method with arithmetic averages (UPGMA) algorithm (73). Cluster analysis was done at 50% Jaccard's similarity coefficient, and distinct clades were considered to represent diverse groups.

**TABLE 3:** Primers and PCR amplification setup for each gene in the present study

Locus	Primer(s)	Thermal cycling conditions	Reference
BOXA1R	5′CTACGGCAAGGCGACGCTGACG3′	7 min at 95°C; 34 × 1 min at 94°C, 1 min at 52.8°C, and 8 min at 65°C; 16 min at 65°C	26
ITS	132F′ (5′CCGGGTTTCCCCATTCGG3′), 1490R′ (5′TGCGGCTGGATCACCTCCTT3′)	3 min at 95°C; 34 × 1 min at 94°C, 1 min at 55°C, and 1 min 45 s at 72°C; 3 min at 72°C	84
16S rRNA	F′ (5′AGAGTTTGATCCTGGCTCAG3′), R′ (5′TACGGTTACCTTGTTACGACTT3′)	4 min at 94°C; 35 × 1 min at 94°C, 1 min at 55°C, and 2 min at 72°C; 10 min at 72°C	85
*gyrB*	343F′ (5′AGCTTGTCCTTSGTCTGCG3′), 1043R′ (5′TTCGACCAGAAYTCCTAYAAGG3′)	2 min at 95°C; 34 × 45 s at 94°C, 30 s at 58°C, and 1 min 30 s at 72°C; 10 min at 72°C	86
*glnII*	13F (5′AAGCTCGAGTACATCTGGCTCGACGG3′), 681R (5′SGAGCCGTTCCAGTCGGTGTCG3′)	2 min at 95°C; 34 × 45 s at 95°C, 30 s at 65°C, and 1 min 30 s at 72°C; 10 min at 72°C	87
*rpoB*	575F (5′ACATCGAGTTCGACGCCAAGG3′), 1054R (5′CATTGACGTGGTCGATGTCG3′)	5 min at 95°C; 20 × 45 s at 95°C, 30 s at 60°C (−0.5°C per cycle) and 1 min 30 s at 72°C; 25 × 30 s at 94°C, 30 s at 55°C, 1 min 30 s at 72°C; 10 min at 72°C	53
*recA*	8F (5′CAACTGCMYTGCGTATCGTCGAAGG3′), 620R (5′CGGATCTGGTTGATGAAGATCACCATG3′)	2 min at 95°C; 34 × 0.4 min at 95°C, 30 s at 67.3°C, and 1 min 30 s at 72°C; 10 min at 72°C	87
*nifH*	28F (5′TACGGNAARGGSGGNATCGGCAA3′), 809R (5′AGCATGTCYTCSAGYTCNTCCA3′)	5 min at 94°C; 20 × 30 s at 94°C, 30 s at 65°C (−0.5°C per cycle), and 1 min 30 s at 72°C; 24 × 30 s at 94°C, 30 s at 55°C, and 1 min 30 s at 72°C; 10 min at 72°C	53

### Determination of diversity index.

To determine the genetic diversity at each experimental site, diversity indices were estimated based on the representative groupings in the dendrogram. Species diversity was computed using the Shannon index (74) as *H*′ = −Σ [(*n*_1_/*N*) ln (*n*_1_/*N*)], where *N* is the total number of all isolates from each experimental site and *n*_1_ is the number of isolates belonging to a particular clade at each experimental site. The bacterial species richness was computed using the Margalef index (75) as *R*_1_ = [(*S* − 1)/ln (*n*)], where *S* is the total number of representative clades at each experimental site and *n* is the total number of isolates in all the clades at each experimental site.

The species evenness was measured using the Pielou index (76) as E_1_ = *H*′/ln(*S*), where *H*′ is the Shannon index and *S* is the total number of representative clades at each experimental site.

### PCR amplification of the 16S-23S rRNA ITS region.

The intergenic transcribed spacer (ITS; 16S-23S rRNA) region of the bacterial genomic DNA was amplified using the respective primer pairs to generate fingerprint patterns. DNA amplification was performed as described for BOX-PCR fingerprinting; a list of primers and thermal cycling conditions are presented in Table 3.

### RFLP analysis of the PCR-amplified products of the 16S-23S rRNA (ITS) region.

The PCR-amplified ITS products were digested using 6-bp-cutting (HindIII), 4-bp-cutting (HaeII), and 5-bp-cutting (HinfI) restriction endonuclease enzymes. The enzyme digestion was carried out by following the manufacturer's instructions (Thermo Scientific, Lithuania). Gel electrophoresis of the digested products was done on a 3% agarose gel at 85 V for 2.5 h. Band sizes were scored against a 100-bp ladder (GeneRuler).

The fingerprint banding patterns generated were analyzed by designating an alphabet to each distinct pattern. The different patterns were then scored based on the presence (1) or absence (0) of the three test endonuclease enzymes' digestion site for each isolate, thus generating a binary matrix. The Jaccard's similarity coefficient (72) was calculated using the similarity matrices, followed by cluster analysis using the UPGMA algorithm (73) with NTSYS pc 2.1 software (USA) (77).

### PCR amplification of the 16S rRNA, *nifH*, and housekeeping genes (*glnII*, *rpoB*, *recA*, and *gyrB*).

The target genes from the rhizobial genomic DNA of each test isolate were amplified using suitable primer pairs and thermal cycling conditions as described for BOX-PCR fingerprinting. All PCR-amplified products (16S rRNA, *nifH*, *glnII*, *rpoB*, *recA*, and *gyrB* regions) were purified with a PCR cleanup kit (New England BioLabs, USA), followed by gene sequencing (Macrogen, The Netherlands).

### Sequence and phylogenetic analysis.

The integrity of each gene sequence was verified using BioEdit 7.0.0 software (78), followed by the determination of their relatedness to type reference strain sequences in the GenBank database (https://www.ncbi.nlm.nih.gov/genbank/) using the BLAST_n_ program. Each isolate sequence was aligned with type reference strain (GenBank database) using MUSCLE (79). Phylogenetic trees were generated from the aligned sequences using the MEGA 6 software program (80), which calculates evolutionary distances using the Kimura 2-parameter model (81). The evolutionary history was inferred using the maximum likelihood method algorithm with 1,000 bootstraps (82). The pairwise sequence identities of single and concatenated gene sequences were calculated using the Bio-edit sequence identity matrix. The conserved, variable, and parsimony nucleotide informative sites were determined for the genes under study (MEGA 6). The sequences were then submitted to GenBank to obtain accession numbers (Table S1).

### Metagenomic analysis of 16S rRNA.

Rhizosphere soil samples of two test cowpea varieties grown at the experimental sites (Muriaze, Ruace, and Sussundenga) were collected and stored at −20°C prior to DNA isolation. Bacterial genomic DNA was extracted from 0.5 g of rhizosphere soil using PowerSoil DNA isolation kit according to the manufacturer's instructions (Mo Bio, USA). Each DNA sample was subjected to PCR amplification using primer pairs that contained adapter-ligated fragments 5′TCGTCGGCAGCGTCAGATGTGTATAAGAGACAGCCTACGGGNGGCWGCAG3′ and 5′GTCTCGTGGGCTCGGAGATGTGTATAAGAGACAGGACTACHVGGGTATCTAATCC3′, thus targeting variable regions V3 and V4 of the 16S rRNA gene (83). The PCR was carried out with 10 to 15 ng DNA in 25-μl reaction volume containing 12.5 μl of 2× KAPA HiFi hotstart ready mix and 5 μl of each primer (1 μM), with the following temperature profile: 95°C for 30 s, 25 cycles of 95°C for 30 s, 55°C for 30 s, and 72°C for 30 s, and then 72°C for 5 min. The PCR-amplified samples were sent to Macrogen, South Korea, for Miseq Illumina paired-end sequencing and analysis.

### Accession number(s).

The sequences of cowpea-nodulating rhizobial isolates used in this study are available in the GenBank database under accession numbers KY941240 through KY941398.

## Supplementary Material

Supplemental material
