# Supplementary material for: Distribution and Phylogeny of Microsymbionts Associated with Cowpea (Vigna unguiculata) Nodulation in Three Agroecological Regions of Mozambique
Source: Appl Environ Microbiol. 2018 Jan 2;84(2):e01712-17. doi: 10.1128/AEM.01712-17 (PMC5752868; doi:10.1128/AEM.01712-17)
Supplement: Supplemental material [file supp_84_2_e01712-17__index.html]

Supplemental material 

# Distribution and Phylogeny of Microsymbionts Associated with Cowpea (Vigna unguiculata) Nodulation in Three Agroecological Regions of Mozambique

## Supplemental material

- Supplemental file 1 -

  GenBank accession number of the sequences used in this study for cowpea-nodulating rhizobial isolates (Table S1); nucleotide information for the gene sequences used in the present study (Table S2); phylogenetic relationships (Fig. S1 to S4).

  PDF, 154K
