# Supplementary material for: Distribution and Phylogeny of Microsymbionts Associated with Cowpea (Vigna unguiculata) Nodulation in Three Agroecological Regions of Mozambique
Source: Appl Environ Microbiol. 2018 Jan 2;84(2):e01712-17. doi: 10.1128/AEM.01712-17 (PMC5752868; doi:10.1128/AEM.01712-17)
Supplement: Supplemental material [file AEM.01712-17_zam002188248s1.pdf]

Distribution and phylogeny of microsymbionts associated with cowpea (*Vigna unguiculata*) nodules across different agro-ecological regions of Mozambique

Ifeoma N Chidebe[1], Sanjay K Jaiswal[2], Felix D Dakora[2]
[1]Department of Crop Sciences, [2]Department of Chemistry, Tshwane University of Technology, Arcadia campus, Pretoria, South Africa

Table S1. GenBank accession number of the sequences used in this study for Cowpea nodulating rhizobial isolates

**Strain**	**16S rDNA**	***glnII***	***gyrB***	***recA***	***rpoB***	***nifH***
TUTVU1	KY941240	KY941263	KY941292	KY941349	KY941379	KY941323
TUTVU3		KY941264	KY941295	-		KY941324
TUTVU5	KY941241	KY941265	KY941293	KY941350	KY941381	KY941325
TUTVU7	KY941242	KY941266	KY941294	KY941351	KY941382	KY941326
TUTVU11	KY941243	KY941267	KY941296	KY941352	KY941383	KY941327
TUTVU13	KY941244	KY941268	KY941297	KY941353	KY941384	KY941328
TUTVU14		KY941269	KY941298	KY941354	KY941385	KY941329
TUTVU21	KY941255	KY941270	KY941299	KY941355	KY941386	KY941330
TUTVU22	KY941245	KY941271	KY941300	KY941356	KY941387	KY941331
TUTVU30		KY941272	KY941301	KY941357	KY941388	KY941332
TUTVU36	KY941246	KY941273	-	KY941358	KY941389	KY941333
TUTVU39	KY941247	KY941274	KY941302	KY941359	KY941390	KY941334
TUTVU42				KY941374	-	KY941335
TUTVU44	KY941248	KY941275	KY941303	KY941360	KY941391	KY941347
TUTVU47			KY941304	KY941361	-	-
TUTVU54		KY941276	KY941305	KY941362	KY941392	KY941348
TUTVU55	KY941249	KY941277	KY941306	KY941363	KY941393	KY941336
TUTVU59		KY941278	KY941307	KY941364	-	KY941337
TUTVU63	KY941256	KY941279	KY941308	KY941365	KY941394	KY941338
TUTVU66		KY941280	KY941309	KY941366	-	KY941339
TUTVU68					KY941395	
TUTVU70	KY941250	KY941281	KY941310	KY941367	KY941375	KY941340
TUTVU77		KY941282	KY941311	KY941368	KY941376	KY941341
TUTVU81	KY941251	KY941283	KY941312	KY941369	KY941377	KY941342
TUTVU86	KY941252	KY941284	KY941314	KY941370	KY941378	KY941343
TUTVU87	KY941253	KY941285	KY941313	KY941371	KY941380	KY941344
TUTVU92			KY941315			KY941345
TUTVU98				KY941371		
TUTVU99	KY941254	KY941286	KY941316	KY941372		KY941346
TUTVU31	KY941257	KY941287	KY941317		KY941396	
TUTVU33	KY941258	KY941288	KY941318		KY941397	
TUTVU40	KY941259	KY941289	KY941319		KY941398	
TUTVU50	KY941260	KY941290	KY941320			
TUTVU67	KY941261	KY941291	KY941321			
TUTVU68	KY941262		KY941322			

Table S2: Nucleotide information on the gene sequences used in the present study

Loci	No. of isolates	Gene sequence nucleotide information (%)				Total	Frequency (%)
		Conserved (C)	Variables (V)	Parsimony-informative(Pi)	Singleton (S)		T/C/A/G
***Bradyrhizobium* Lineage**							
16S rRNA	54	449 (84.08)	85 (15.92)	14 (2.62)	71 (13.30)	534	20.51/21.25/25.90/32.33
*gln*II	63	149 (61.07)	95 (38.93)	77 (31.43)	18 (7.38)	244	19.26/31.97/18.03/30.74
*gyr*B	58	162 (45.25)	196 (54.75)	112 (31.28)	84 (23.46)	358	19.83/30.17/16.48/33.52
*rpo*B	53	123 (46.24)	103 (37.72)	55 (20.68)	48 (18.05)	226	14.25/32.66/20.79/32.30
*rec*A	62	180 (59.80)	121 (40.20)	90 (29.90)	31 (10.30)	301	16.61/34.22/16.61/32.56
*gln*II+*gyr*B+*recA*+*rpo*B	38	648 (57.44)	480 (42.55)	290 (25.70)	190 (16.84)	1128	17.9/31.9/17.8/32.4
***Rhizobium* Lineage**							
16S rRNA	59	457 (80.32)	112 (19.68)	72 (12.65)	40 (7.03)	569	20.70/20.78.26.01/35.50
*gln*II	42	245 (61.40)	154 (38.60)	124 (31.08)	84 (23.46)	358	19.83/30.17/16.48/33.52
*gyr*B	29	241 (41.87)	336 (58.23)	257 (44.54)	79 (13.69)	577	22.7/29.4/17.7/30.2
*rpo*B	25	92 (42.20)	126 (57.53)	84 (38.99)	42 (19.27)	218	15.2/31.1/23.2/30.4
*gln*II+*gyr*B	18	558 (57.17)	418 (42.83)	298 (30.53)	120 (12.27)	976	20.75/30.97/18.46/29.81
*gln*II+*gyr*B+*rpo*B	16	663 (55.53)	531 (44.47)	361 (30.23)	170 (14.23)	1194	16.62/31.02/19.34/30.02
*nif*H	58	172 (62.77)	102 (37.22)	88 (32.11)	14 (5.10)	274	19.8/33.8/20.2/26.2

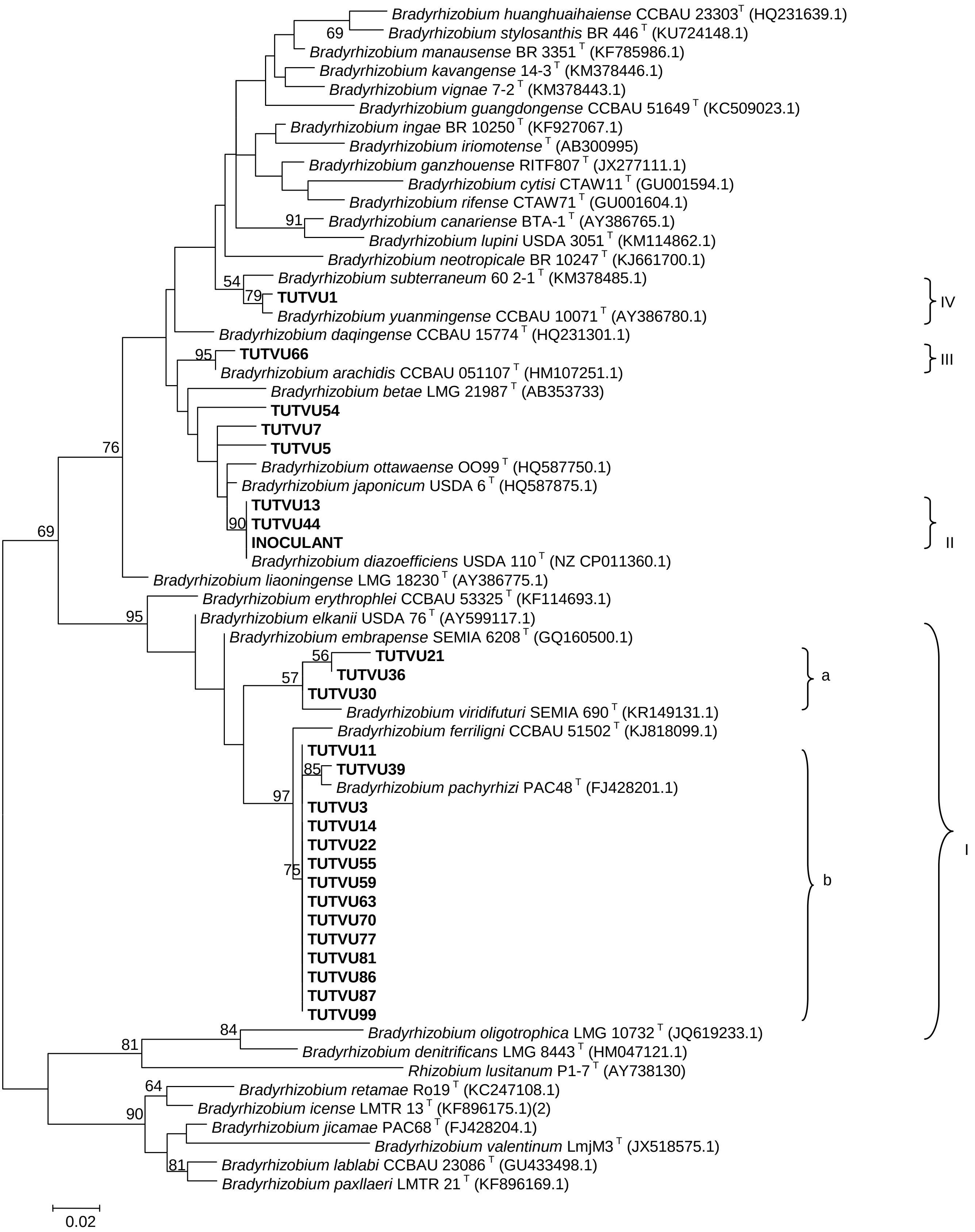

Fig S1a Phylogenetic relationships test isolates of cowpea root nodules and reference *Bradyrhizobium* type strains (NCBI GenBank) based on the *gln*II gene sequences. Bootstrap values are indicated at the nodes

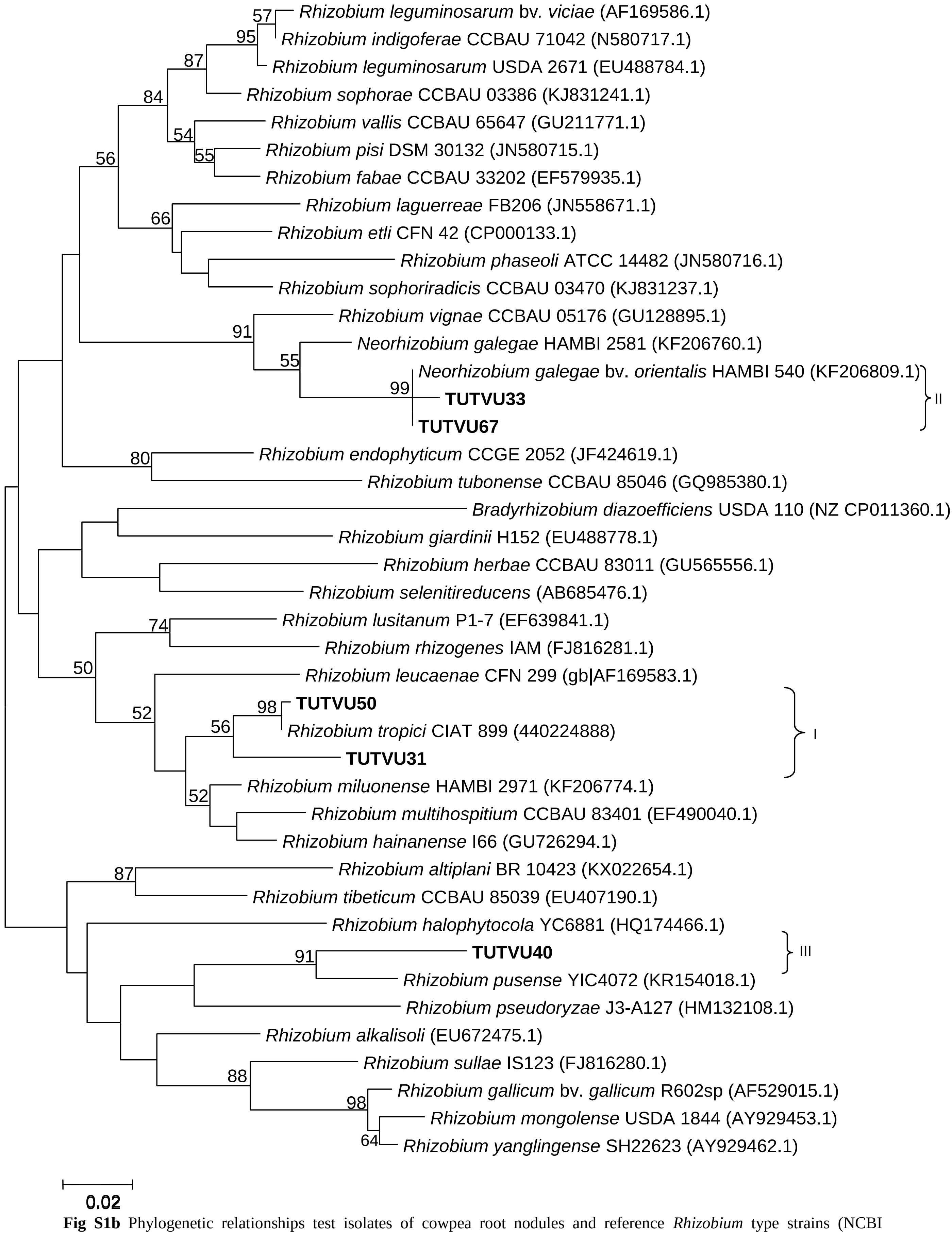

Fig S1b Phylogenetic relationships test isolates of cowpea root nodules and reference *Rhizobium* type strains (NCBI GenBank) based on the *gln*II gene sequences. Bootstrap values are indicated at the nodes

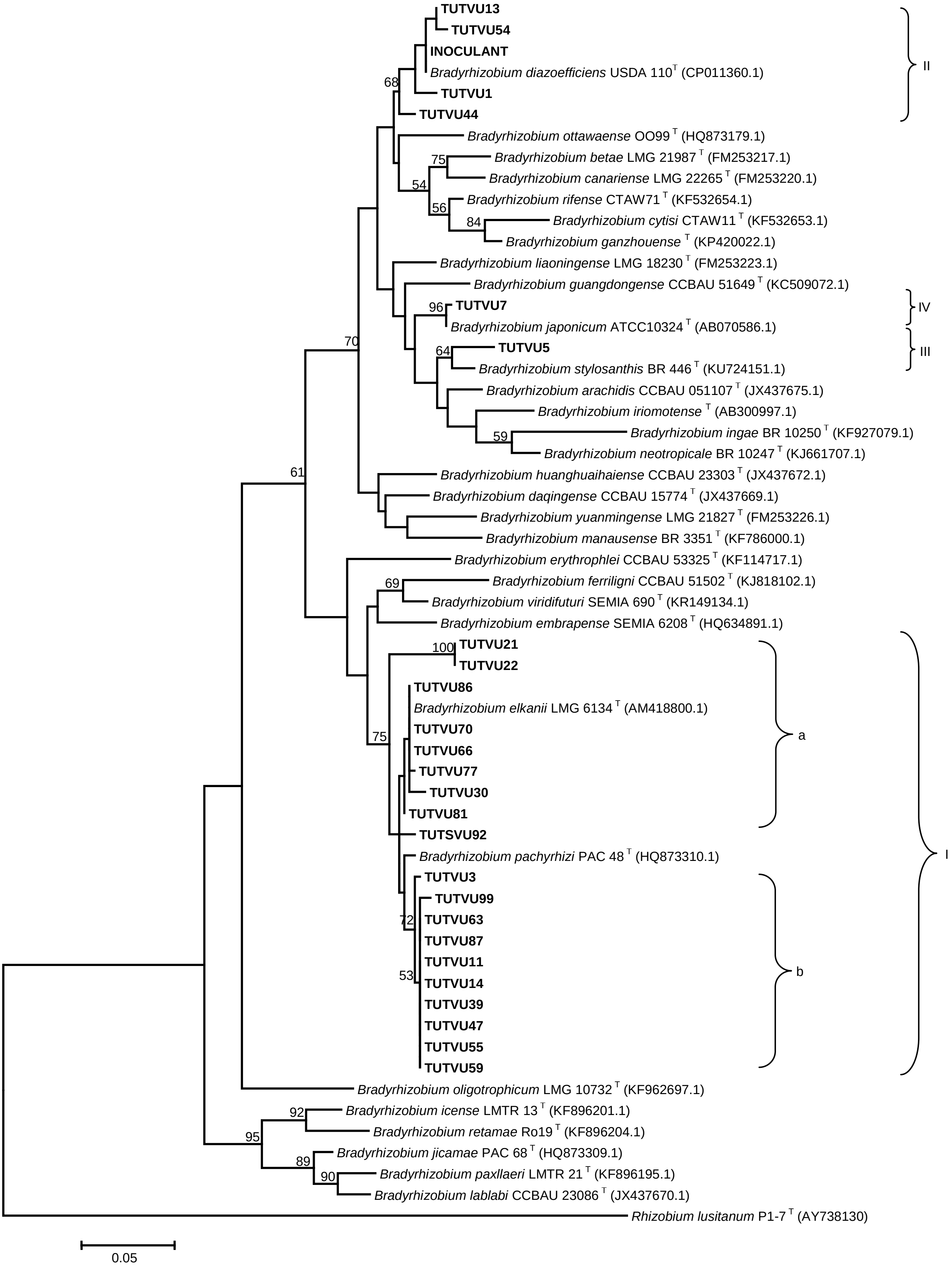

Fig S2a Phylogenetic relationships between test isolates of cowpea root nodules and reference *Bradyrhizobium* type strains (NCBI GenBank) based on their *gyr*B housekeeping gene sequences. Bootstrap values are indicated at the nodes

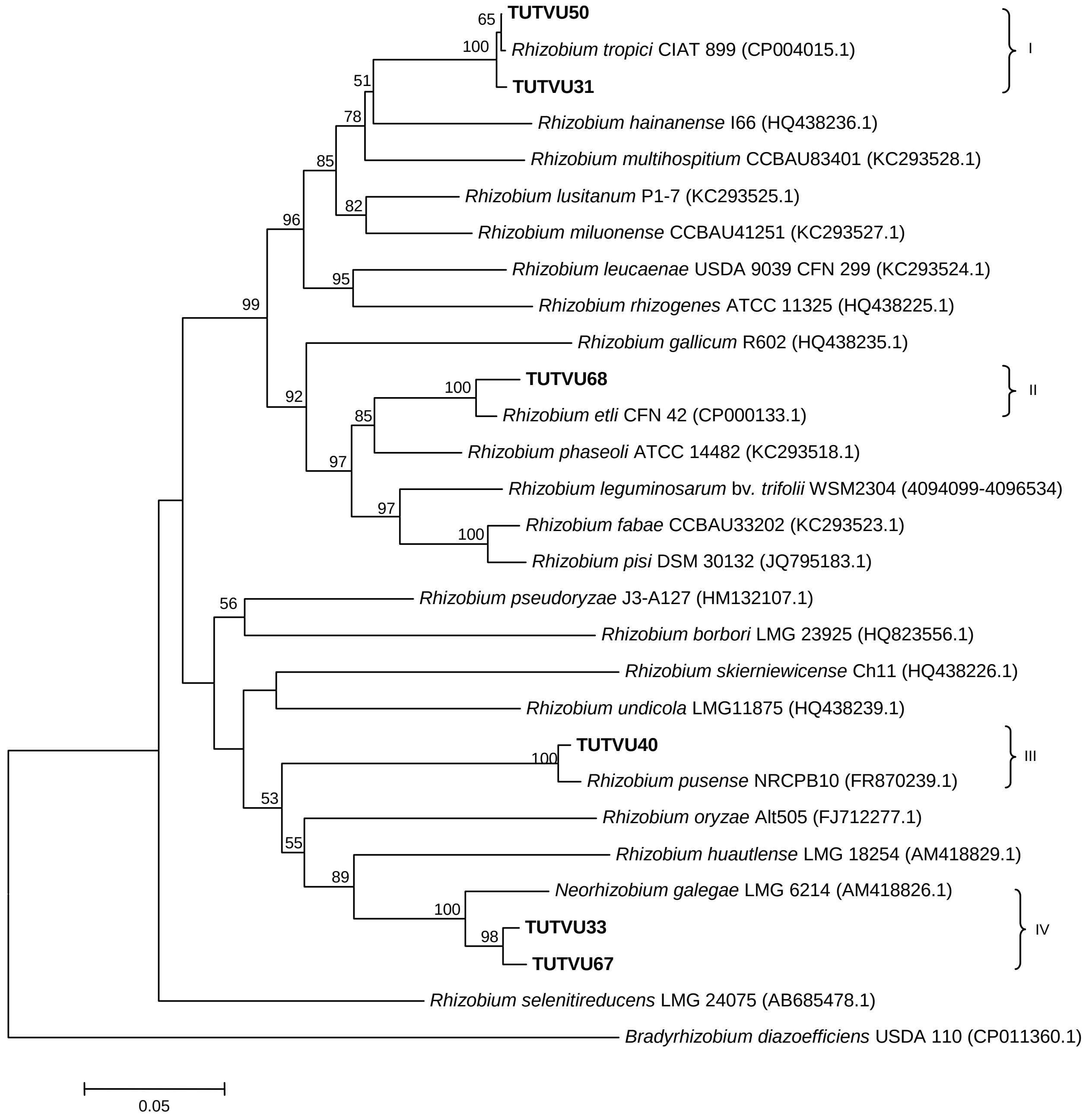

Fig S2b Phylogenetic relationships between test isolates of cowpea root nodules and reference *Rhizobium* type strains (NCBI GenBank) based on their *gyr*B housekeeping gene sequences. Bootstrap values are indicated at the nodes.

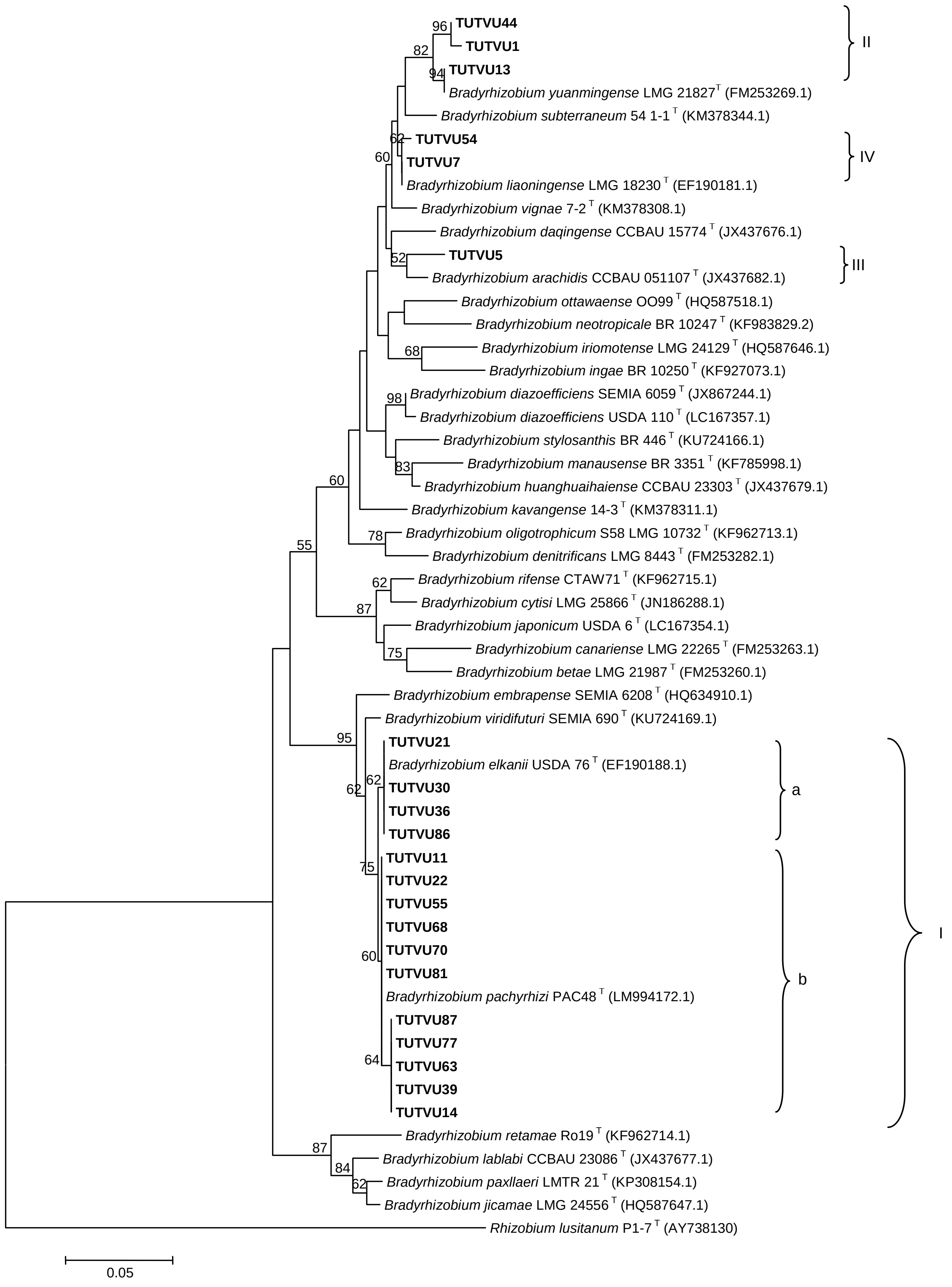

Fig S3a Phylogenetic relationships between test isolates of cowpea root nodules and reference *Bradyrhizobium* type strains (NCBI GenBank) based on their *rpo*B gene sequences. Bootstrap values are indicated at the nodes.

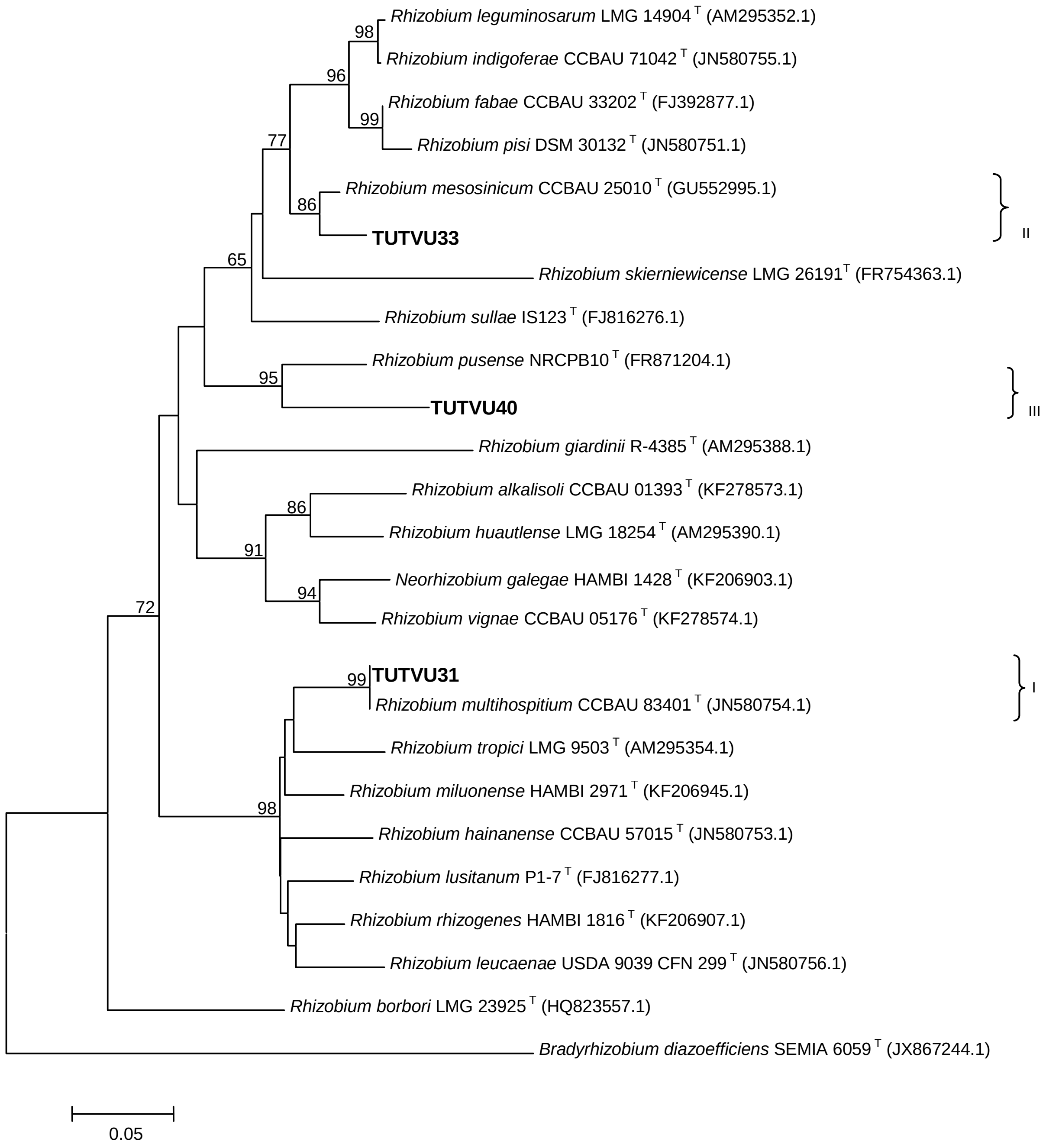

Fig S3b Phylogenetic relationships between test isolates of cowpea root nodules and reference *Rhizobium* type strains (NCBI GenBank) based on their *rpo*B gene sequences. Bootstrap values are indicated at the nodes.

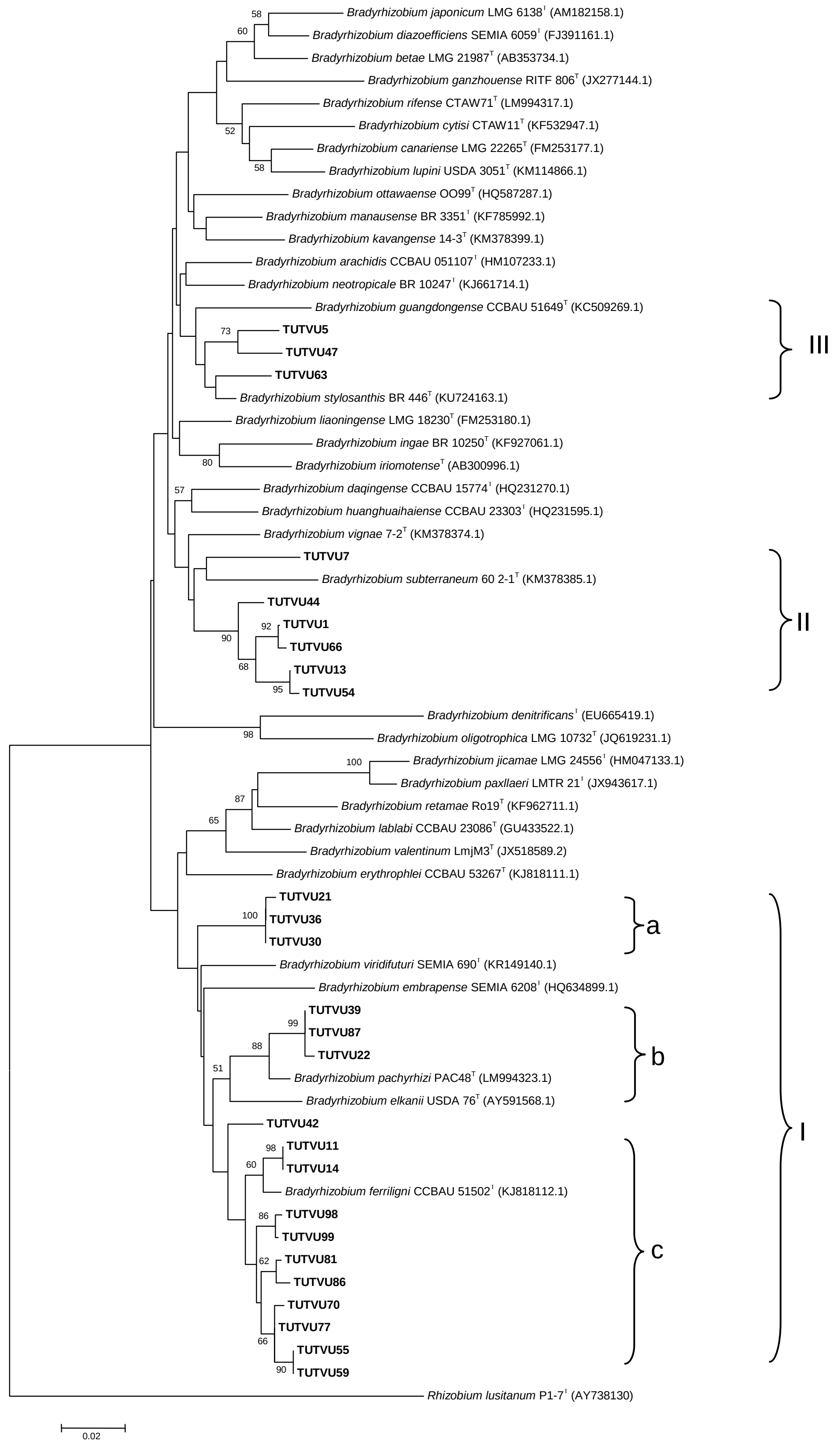

Fig S4 Phylogenetic relationships between test isolates of cowpea root nodules and reference *Bradyrhizobium* type strains (NCBI GenBank) based on *rec*A gene sequences. Bootstrap values are indicated at the nodes
